# CELF1 promotes aerobic glycolysis and an aggressive phenotype in ER-positive breast cancer via GLUT1 regulation

**DOI:** 10.3389/fgene.2025.1687066

**Published:** 2025-11-12

**Authors:** Jinyu Li, Ning Wang, Jianlei Bi, Meihua Guo, Bingbing Xu, Gena Huang

**Affiliations:** 1 Department of Medical Oncology, The Second Hospital of Dalian Medical University, Dalian, Liaoning, China; 2 Institute for Genome Engineered Animal Models of Human Diseases, Dalian Medical University, Dalian, Liaoning, China

**Keywords:** RBPs, CELF1, breast cancer, GLUT1, glycolysis

## Abstract

**Introduction:**

RNA-binding proteins (RBPs) shape post-transcriptional programs in cancer, yet subtype-specific roles in breast cancer remain unclear. We evaluated whether CUGBP Elav-like family member 1 (CELF1), an RBPs with prognostic relevance in luminal A (ER-positive) breast cancer, drives malignant phenotypes via glycolytic reprogramming through glucose transporter 1 (GLUT1).

**Methods:**

We surveyed 1,337 RBPs across TCGA to identify luminal A prognosis-related candidates using Cox models and random-forest ranking, then validated CELF1 biologically. Functional assays combined CELF1 knockdown in ER-positive cells (MCF7, T47D) and overexpression in HER2-positive cells (SKBR3, HCC1954), RNA-seq with differential expression and GSEA, qPCR,western blot, migration, colony assays, IHC in clinical tissues, and a nude-mouse xenograft with the GLUT1 inhibitor BAY-876.

**Results:**

Cox and random-forest analyses prioritized CELF1 among prognosis-related RBPs in luminal A tumors; high CELF1 associated with poorer survival and was overexpressed in breast cancer versus normal tissue. CELF1 modulation bidirectionally altered glycolytic programs and malignant traits: CELF1 loss reduced proliferation, colony formation, migration, and xenograft growth, whereas overexpression enhanced these phenotypes. RNA-seq and enrichment analyses highlighted suppression of glycolysis pathways upon CELF1 loss; GLUT1 (SLC2A1), HK2, and G6PD were consistently downregulated at mRNA and protein levels after CELF1 knockdown and upregulated with CELF1 overexpression. In vivo, combining CELF1 knockout with BAY-876 further curtailed tumor growth and proliferation markers.

**Conclusion:**

CELF1 promotes aerobic glycolysis and aggressive behavior in ER-positive breast cancer, at least partly by regulating GLUT1. These findings reveal RBP-driven metabolic reprogramming in luminal A disease and nominate the CELF1–GLUT1 axis as a potential therapeutic vulnerability.

## Introduction

RNA-binding proteins (RBPs) have long been a subject of interest in the field of oncology due to their capability to alter the localization, stability, and alternative splicing of transcripts that code for a wide range of known tumor suppressors and oncogenes (Hentze et al., 2018; Gerstberger et al., 2014; Tao et al., 2024). In addition, extensive sequencing analysis has revealed a plethora of mutations, mRNA expression changes, and copy number variations (CNVs) in numerous RBPs across a variety of tumor types, including, yet not confined to, brain, lung, kidney, colon, and breast carcinomas (Corley et al., 2020; Van Nostrand et al., 2020; Massey et al., 2024). However, characterizing the contributions of distinct RBPs in the context of malignant transformation is a formidable obstacle. A recent survey of human cells has identified over 1,300 human RNA-binding proteins, and many remain functionally uncharacterized (Hentze et al., 2018).

In breast cancer, RBPs have the potential to promote malignant phenotypes and present therapeutic opportunities (Massey et al., 2024; Wang et al., 2024). Breast tumors exhibit extensive mRNA-level alterations; RBPs are positioned to shape these expression programs. Nonetheless, their subtype-specific roles remain incompletely defined. CUGBP Elav-like family member 1 (CELF1) belongs to the CELF/Bruno-like RNA-binding protein family, which is involved in diverse cellular processes (Dasgupta and Ladd, 2012). CELF1, primarily acknowledged as a vital regulator in the development of myotonic dystrophy type 1 (DM1) disease (Lutz et al., 2023), has now been associated with liver dysfunction (Tan et al., 2022; Ye et al., 2024) and specific cancer types (Xia et al., 2015; Wang et al., 2016; Talwar et al., 2013; Huang et al., 2020), highlighting its multifaceted roles. Furthermore, CELF1 is associated to controlling genes related to cell cycle regulation, apoptosis, and DNA damage response. Dysregulation of CELF1 can affect the expressions of these genes, leading to uncontrolled cell growth and reduced cell death, which are characteristic features of cancer. It is worth highlighting that research is currently delving into the specific ways in which CELF1 is involved in driving cancer development and progression, and the specific roles of CELF1 may vary depending on the cancer type and context.

It is well known that the dysregulated metabolism of glucose and lipids is closely linked to cancer incidence and aggressiveness (Braun et al., 2011; Faulds and Dahlman-Wright, 2012). Not surprisingly, glycolysis is an important feature of tumor occurrence and progression. Glucose transporter 1 (GLUT1), a member of the glucose transporter family, is crucial in controlling the transport of glucose through the cellular membrane (Deng et al., 2014). The movement of glucose through the cell membrane is not only a critical process that regulates the pace of glycolysis but also represents the initial phase of glucose metabolism. Numerous research studies have demonstrated that GLUT1 exerts a notable influence on different types of cancer, such as lung, breast, and renal cancers. Its participation manifests in promoting cell growth and metastasis, while concurrently suppressing apoptosis (Oh et al., 2017; Chan et al., 2011; Zhou et al., 2024).

In this study, we analyzed clinical datasets to provide an impartial assessment of the genetic conditions (mutations, amplifications, deletions, and translocations) of all recognized RBPs in breast cancers. Among RBPs in luminal breast cancer, CELF1 emerged as a leading contributing factor. CELF1 is particularly relevant in the context of alternative splicing, which is important in generating protein diversity (Timchenko et al., 1999; Timchenko et al., 2001). In the previous study, we explored the influence of CELF1 on the alternative splicing of INSR and observed a shift in its oncogenic effects in breast cancer; these effects were found to differ among various molecular subtypes (Huang et al., 2020). Few studies have been conducted on the correlation between CELF1 and metabolism thus far. In the context of this study, we observed that CELF1 is expressed and functional across diverse molecular subgroups of breast cancer; additionally, by modulating the expression of GLUT1, CELF1 stimulates aerobic glycolysis in breast cancer cells.

## Materials and methods

### Dataset source

Data on copy number alterations and mutations for 33 types of tumors were acquired from the TCGA database via UCSC Xena (Goldman et al., 2020). This also included mRNA expressions and clinical information for 10,289 patient samples from the TCGA PanCancer dataset, also sourced from UCSC Xena (Weinstein et al., 2013). For the comparative analysis of tumor versus normal samples, only 19 of the 33 tumor categories, each with at least five pairs of tumor and normal samples, were selected for inclusion.

### Human RBP catalog

A catalog of 1,393 RBP genes, curated by Matthias W. Hentze et al. in 2017, was downloaded (Hentze et al., 2018). These genes were meticulously described according to Ensembl version 111 and categorized into various subclasses based on their RNA-binding domains, following the Pfam classification system. After evaluating the curated RBPs for duplicates, we eliminated any redundancies. By integrating data from TCGA, we identified a total of 1,337 RNA-binding proteins.

### CNV and SNV analyses

In CNV data, a value of 2 indicates amplifications, while a value of −2 signifies deep deletions (Gu and Hübschmann, 2023). For single-nucleotide variant (SNV) data, only non-silent mutations such as missense, nonsense, frame shift deletions, splice site alterations, frame shift insertions, in-frame deletions, and nonstop mutations were included. The study calculated the ratios of SNV and CNV for every type of tumor. Moreover, the comprehensive somatic changes across tumors were visualized in the OncoPrint plot, created with the ComplexHeatmap package.

### Establishing the RBP potential index

An index to quantify RBP levels was developed using expression data from 1,337 RBP genes (Hänzelmann et al., 2013). The enrichment score (ES) for gene sets regulating RBP was determined through single-sample gene set enrichment analysis (ssGSEA) using the GSVA package. The RBP potential index, representing normalized differences in ES, was then used to computationally analyze RBP levels/trends in tissue samples.

### Identification of prognosis-related RBPs

An examination of the connection between RBP expression and overall survival (OS) among individuals diagnosed with luminal A breast cancer was conducted through univariate Cox proportional hazards regression analysis within the TCGA dataset, selecting RBPs with a Benjamini–Hochberg (BH) FDR–adjusted P < 0.05. Subsequently, the randomForestSRC package’s rfsrc function refined this selection of RBPs (Taylor, 2011). We trained a random survival forest with ntree = 1,000 and computed permutation-based variable importance (VIMP) with nrep = 1,000 Monte-Carlo repetitions to rank genes. Through a random survival forest algorithm, an iterative process was employed to progressively reduce the gene set, eliminating the least significant 25% of RBPs at each step. At every iteration, 1,000 trees were cultivated, with the number of RBPs randomly selected at each node set to the square root of the total number of input nodes for that tree. Adjustments were made to the class weights due to the imbalance between patients with good and poor prognosis. The out-of-bag samples provided an estimate of the generalization error. Ultimately, this process resulted in the selection of six RBPs.

### DepMap analysis

In the DepMap analysis, gene effect scores derived from CRISPR knockouts and mRNA expression data were sourced from the DepMap 23Q4 release via the Broad Institute’s DepMap portal (Pacini et al., 2024). The analysis excluded cell lines identified as fibroblast, teratoma, unknown, engineered, or non-cancerous. A linear model was applied using R to explore the relationship between the effect of the CELF1 gene and mRNA expression. Correlation coefficients were used for preliminary ranking. The analysis utilized the GSEA Preranked algorithm for Hallmark gene sets from MSigDB (version 2023.2), calculating normalized enrichment scores (NESs) based on a pre-ranked list of genes. This was achieved through 1,000 permutations, with significance established at an FDR q-value below 0.05 (Tsherniak et al., 2017; Ghandi et al., 2019).

### Samples and clinicopathological data

From January 2008 to January 2014, we collected 94 breast cancer specimens, along with paired adjacent non-cancerous breast tissue, through surgical resection at the Second Hospital of Dalian Medical University. Patients were excluded if they 1. had a history of other malignancies, 2. received neoadjuvant chemotherapy or radiotherapy, or 3. had incomplete clinical records. The study’s selection criteria included the following: 1. histopathological confirmation of ER-positive, PR-positive, and HER2-negative breast cancers, 2. post-operative analysis of at least 15 lymph nodes, 3. the ability to uniformly treat tumor samples with the CELF1 antibody, and 4. the presence of comprehensive medical records for each patient. Tissue samples were systematically randomized using a computer-generated sequence to ensure unbiased allocation for experimental analyses. Clinical data collection included patient age, menopausal status (pre-/post-menopausal), and standard pathological parameters. The study’s research protocol received ethical approval from the Ethics Committee of the Second Hospital of Dalian Medical University.

### Cell culture

Breast cancer cell lines MCF7, T47D, SKBR3, HCC1954, MDA-MB-436, and MDA-MB-231 were obtained from the American Type Culture Collection (ATCC, Manassas, United States). These cell lines were grown in RPMI 1640 medium (Gibco, United States) enriched with 10% fetal bovine serum (FBS) and kept at 37 °C in a 5% CO2 incubator. MCF-7 and T47D are characterized by their estrogen receptor positivity, while SKBR3 and HCC1954 are noted for Her-2 receptor overexpression. Conversely, MDA-MB-436 and MDA-MB-231 are identified as triple-negative breast cancer cell lines due to their lack of estrogen, progesterone, and Her-2 receptor expressions.

### shRNA constructs

Two shRNA hairpins were designed to target human CELF1 (shCELF1-1 or shCELF1-2), along with a control shRNA (shNC), into the LV-3 (pGLVH1/GFP+Puro) plasmid. These plasmids were labeled as shCELF1-1, shCELF1-2, or shNC, respectively (GenePharma, Suzhou, China). After transfection, cells were collected at 24 h and 48 h for PCR and Western blot analyses, respectively. The LV-3-CELF1, shCELF1-1, and shCELF1-2 plasmids underwent sequencing for validation of their orientation and integrity. CELF1 shRNA#1: 5′-GGT​TGA​ATG​CAA​TGC​AGT​TAC-3′; CELF1 shRNA#2: 5′-GCA​GGA​ATG​GCT​GCT​TTA​AAT-3’.

### Plasmid construction

The open reading frame (ORF) of CELF1 was amplified using PCR and subsequently cloned into a pcDNA3.1 vector (sourced from GenePharma, Suzhou, China) at the EcoRI and BamHI sites. The transfection protocol previously employed for shRNA introduction was also used for this process.

### Cell proliferation assay

Following transfection with shRNA or plasmids, cells were plated at a density of 5,000 cells per well in a 96-well plate. We utilized the CCK8 assay kit from Abbkine (Wuhan, China) to evaluate cell proliferation, following the manufacturer’s instructions. In summary, 10 μL of CCK8 solution was added to each well and incubated at 37 °C for 2 h. Subsequently, the absorbance of each sample was gauged at 450 nm using a Thermo (United States) microplate reader.

### Colony formation assay

After being suspended in a serum-free medium and transfection with shRNA or plasmids, the cells were introduced into 6-well plates at a density of 1,000 cells per well, optimal for subsequent analyses. Over 2 weeks, colonies were allowed to form and expand. Post-incubation, colonies were fixed using formaldehyde and stained with crystal violet for visualization. Colony images were captured, and the colony count per well was quantified using ImageJ software.

### Transwell assay

For the cell migration study, transwell chambers with 8-μm pores (Costar) were employed. Cells transfected with shRNA or plasmids were grown to 75%–80% confluence before initiation of the migration assay. Cells were then trypsinized, rinsed with phosphate-buffered saline (PBS), and resuspended in a serum-free medium. Subsequently, 100 μL of the cell suspension, containing 10 × 10^4^ cells, was placed into the transwell’s upper chamber, while the lower chamber was filled with medium supplemented with FBS to encourage migration. Post-migration, cells remaining on the upper surface were removed with cotton swabs. Migrated cells, on the other hand, were fixed with formaldehyde and stained with crystal violet for better visualization. Photographs of six different ×10 fields per membrane were taken to count the migratory cells. The average number of migratory cells was calculated based on the mean values from triplicate assays for each experimental condition.

### RNA-seq library generation

Total RNA was extracted from 5 × 10^6^ CELF1 knock-out and wild-type MCF7 cells using the TRIzol method, following the manufacturer’s guidelines. The extracted RNA was assessed using the Agilent RNA 6000 Assay Kit on an Agilent Bioanalyzer 2100 system. Library preparation followed the TruSeq RNA Sample Preparation Kit (Illumina) protocol. The libraries underwent paired-end sequencing (150 bp) on an Illumina NovaSeq platform.

### Differential gene expression and enrichment analyses

RNA-seq reads underwent gene expression processing, starting with quality control and adapter removal using FastQC, followed by alignment to the GRCh38 reference genome and initial quantification with STAR (Dobin et al., 2013). Transcripts with a minimum count of ≥10 were prefiltered, and differential expression analysis was conducted using the DESeq2 package to compare wild-type with knock-out samples (Love et al., 2014). Significant alterations were identified with a threshold of |Log2-fold change| ≥1 and an adjusted P-value ≤ 0.05. For enrichment analysis, the clusterProfiler package was utilized, applying gene sets from GO and the Hallmark category in the Molecular Signatures Database (MSigDB) (Wu et al., 2021; Subramanian et al., 2005). The GseaVis package was employed to effectively present the GSEA results (Liberzon et al., 2015).

### Western blot

Total cell lysates underwent protein extraction via treatment with radio-immunoprecipitation assay (RIPA) buffer; protein concentrations were quantified using a BCA Protein Assay Kit (Thermo Fisher, A55865). Proteins were then resolved on a 12% SDS-PAGE gel and transferred to nitrocellulose membranes. These membranes were probed with monoclonal antibodies targeting CELF1, GLUT1, cyclin D1, cyclin B1, c-Myc, HK, G6PD, and GAPDH (Proteintech, Wuhan, China), as well as Bcl-2 and BAX (Abbkine, United States). Protein bands were detected using the SuperLumia ECL HRP Substrate Kit (Abbkine, United States). The bands were quantitatively analyzed using Quantity One software (Bio-Rad, Hercules, CA, United States), and band intensities were normalized to GAPDH using ImageJ.

### RNA isolation and reverse-transcriptase PCR quantification process

The total RNA from cultured cells was isolated using the TRIzol reagent (Takara), following the manufacturer’s instructions. Complementary DNA (cDNA) was then generated from the extracted total RNA or purified small RNAs utilizing the TransScript One-Step gDNA Removal and cDNA Synthesis SuperMix (TransGen Biotech, Beijing, China) as per the provided guidelines. PCR was performed using Taq polymerase acquired from Takara and specialized primers designed to aim at CELF1 (forward: 5′-ACA​TCC​GAG​TCA​TGT​TCT​CTT​CG-3′ and reverse: 5′-CAT​TGC​CTT​GAT​AGC​CGT​CTG-3′), GLUT1(SLC2A1) (5′- GGC​CAA​GAG​TGT​GCT​AAA​GAA-3′ and reverse: 5′- ACA​GCG​TTG​ATG​CCA​GAC​AG-3′), HK2 (forward: 5′- GAG​CCA​CCA​CTC​ACC​CTA​CT-3′ and reverse: 5′- CCA​GGC​ATT​CGG​CAA​TGT​G-3′), G6PD (forward: 5′- CGA​GGC​CGT​CAC​CAA​GAA​C -3′ and reverse: 5′- GTA​GTG​GTC​GAT​GCG​GTA​GA -3′), and glyceraldehyde 3-phosphate dehydrogenase (GAPDH) (forward: 5′- GGA​GCG​AGA​TCC​CTC​CAA​AAT-3′ and reverse: 5′- GGC​TGT​TGT​CAT​ACT​TCT​CAT​GG-3′). For normalization, GAPDH was utilized as an internal reference gene. The PCR protocol involved an initial denaturation at 94 °C for 3 min, followed by 35 cycles of denaturation at 94 °C for 30 s, annealing at 55 °C for 45 s, and extension at 72 °C for 1 min, concluding with a final extension at 72 °C for 15 min. The PCR products were visualized on 2% agarose gels stained with ethidium bromide (10 mg/mL) under UV light. The TransStart Tip Green qPCR SuperMix (Transgene Biotech) was employed for real-time PCR analysis on an ABI 7900HT FAST Real-Time PCR System (Applied Biosystems); relative mRNA expression was calculated using the 2^−ΔΔCT^ method, normalized to GAPDH.

### Immunohistochemistry

Ninety-four formalin-fixed, paraffin-embedded primary breast tumor samples, along with adjacent breast tissue, were analyzed using immunohistochemistry. These samples were provided by the Department of Breast Surgery at the Second Hospital of Dalian Medical University, following thorough histopathological and clinical assessments. IHC was also conducted on tumors from nude mice. Deparaffinization, rehydration, and antigen retrieval procedures were performed on the tissue sections via microwave treatment in 10 mM citrate buffer (pH 6.0) for 10 min. Sections were treated with 0.3% hydrogen peroxide for 15 min and then incubated with primary antibodies against human CELF1, GLUT1, and Ki-67 (1:100 dilution) at 4 °C overnight. Afterward, the samples were incubated with HRP-conjugated immunoglobulin for 30 min. The detection was completed using 3′-3′ diaminobenzidine (DAB) as the chromogenic substrate.

### Immunofluorescence

MCF7 and SKBR3 cells were fixed for immunofluorescence analysis using 4% paraformaldehyde and permeabilized with 0.5% Triton X-100. The cells were then incubated with goat serum before being exposed to a primary antibody against GLUT1 overnight at 4 °C. Subsequently, the samples were exposed to a secondary antibody and DAPI for nuclear staining, then mounted, and visualized using a Nikon Eclipse Ni-E fluorescence microscope.

### Nude mouse subcutaneous tumor formation experiment

To investigate CELF1’s function *in vivo*, female nude BALB/c mice from the Model Animal Research Center of Nanjing Medical University were subcutaneously injected in the axillary fossa with 4 × 10^6^ T47D cells. These cells were previously transfected with either the control plasmid (shNC) or the CELF1-targeting plasmid (shCELF1-1). Tumor growth was monitored, and when tumors reached approximately 60 mm^3^ in volume, their size was recorded every 4 days. No significant differences in tumor volume were observed either between or within the experimental groups. The mice received oral 3 mg/kg/day of BAY-876 for 3 consecutive days. The pharmacokinetic characteristics of BAY-876 were then assessed using established methods in this subcutaneous tumor model (Siebeneicher et al., 2016). Tumor and body weights of the mice were measured at 3-day intervals, with the study concluding on day 21. At this point, mice were anesthetized with isoflurane (2%–3% in oxygen) and euthanized, and then the tumors were collected for further analysis. The formula V = (W^2^ × L)/2 was used to calculate the tumor volume, where V is the volume, W is the width, and L is the length. All animal procedures were performed in accordance with the National Institutes of Health Guide for the Care and Use of Laboratory Animals, ARRIVE guidelines 2.0, and institutional regulations of the Second Hospital of Dalian Medical University. The study protocol was approved by the Ethical Committee of the Second Hospital of Dalian Medical University (Approval No: 2019-ETH-102).

### Statistical analysis

Statistical analyses were conducted using GraphPad Prism v9.5.1 or R v4.2.1. Data with n < 15 were analyzed using Mann–Whitney U (two groups)/Kruskal–Wallis tests (>2 groups). Parametric tests were reserved for larger samples (n ≥ 30) with confirmed normality; a two-tailed Student’s t-test facilitated the comparison between two groups, and ANOVA was utilized for assessing differences among three or more groups. Normality was verified using the Shapiro–Wilk tests for all continuous variables (*P* > 0.05). Outcomes were expressed as mean ± standard deviation, indicating both the central tendency and variability of the data. These results were based on at least three independent experiments, confirming their consistency and reliability.

## Results

### Genetic alterations of RBPs in pan-cancers

**FIGURE 1:**
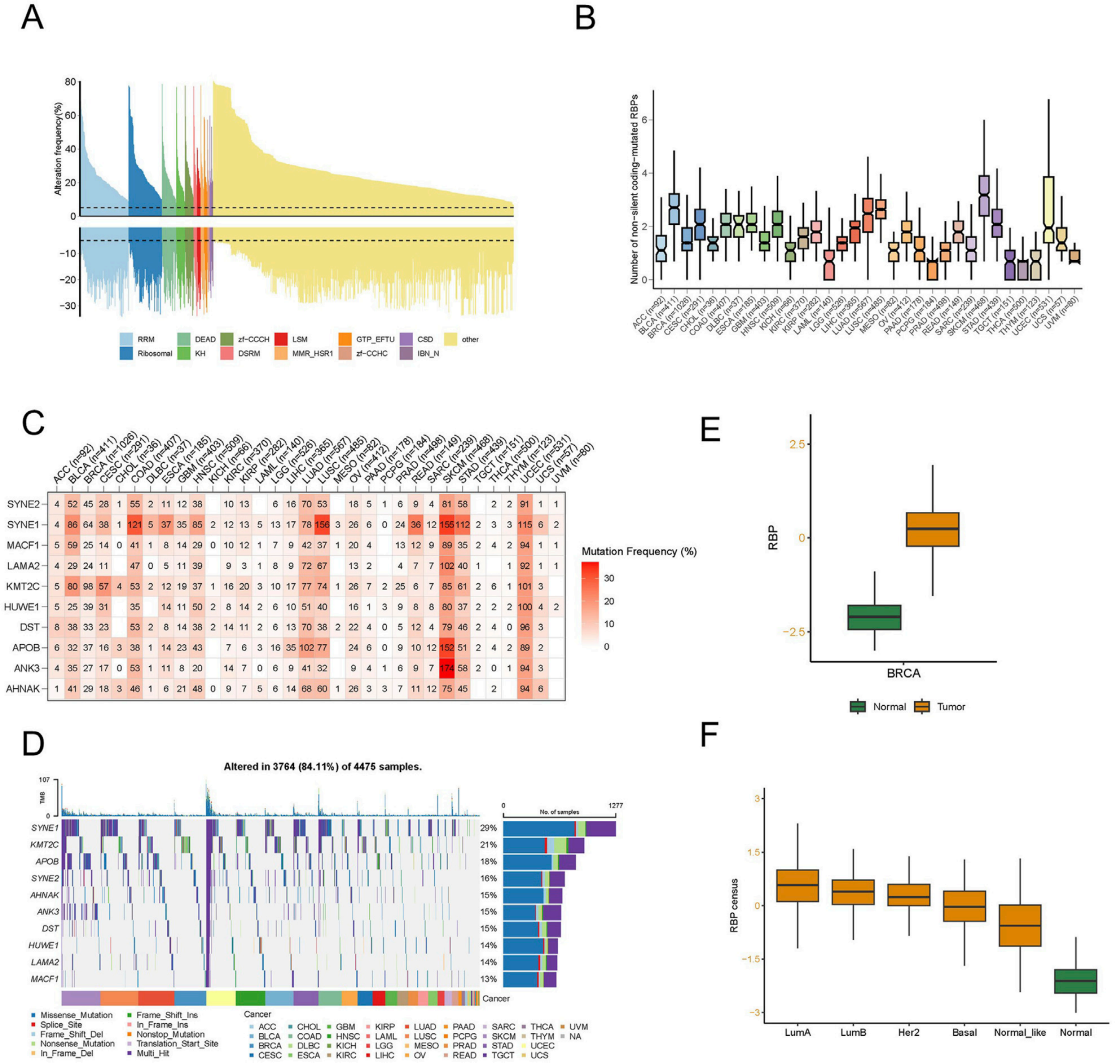
A distinct landscape of RNA-binding proteins (RBPs) in pan-cancer. **(A)** Histogram illustrates the occurrence of somatic copy number changes (deletions or amplifications) in breast cancer for all identified RBPs, categorized based on their RNA-binding domains using Pfam nomenclature. Data were obtained from the breast cancer TCGA database through UCSC XENA. **(B)** The count of non-silent coding-mutated RBPs in each sample across the 33 cancer types. The data underwent preprocessing involving log2-based conversion. **(C)** Mutation frequency of top 10 RBP-responsive genes. Numbers indicate the count of samples where the specific mutated gene is present in a particular cancer type. “0” signifies the absence of mutations in the coding region of the gene, while the absence of any number denotes no mutations in any area of the gene. **(D)** Oncoplot illustrating the distribution of mutations in RBP-responsive genes and categorization based on SNV types. **(E)** The different RBP potential index between tumor and normal tissues in breast cancer. **(F)** The different RBP potential index among different molecular subtypes of breast cancer.

To ascertain the extent of alterations of RBPs in human cancers, we explored 1,337 known human RBPs across the entire Cancer Genome Atlas (TCGA) and analyzed factors influencing protein CNVs and mutations. Somatic copy number analysis (SCNA) is frequently reported in cancer genomes (Beroukhim et al., 2010; Zack et al., 2013) and could potentially hamper the expressions of crucial genes that facilitate carcinogenesis. We assessed the extent of CNVs (both amplifications and deletions) among RBPs in various cancers. Generally, we found high rates of copy number amplification in most cancers, with more than 5% of samples showing this trend, except for RBPs in thyroid cancer (THCA), which had a low SCNA frequency (Supplementary Figure S1A). When considering both amplifications and deletions, breast cancer (BRCA) had a relatively higher frequency of genomic changes in RBPs (Figure 1A; Supplementary Figure S1B). Additionally, mutations play a significant role in cancer (Kandoth et al., 2013; Lawrence et al., 2013). We, therefore, analyzed the mutation patterns of these 1,337 RBPs across 33 types of cancer, identifying 135,820 somatic mutations in 10,289 tumor samples. Remarkably, 91.1% of these samples had at least one RBP mutation. The number of non-silent mutations within RBPs varied widely within the same cancer type, with some samples showing only a few mutations and others presenting hundreds (Figure 1B).

We examined SNP data related to RBPs to assess mutation frequencies and types across different cancer subtypes. In particular, we focused on the top 10 RBPs exhibiting the highest frequencies of non-silent mutations, with SNV rates varying from 10% to 37% in UCEC, SKCM, and COAD (Figure 1C). Among these cancers, the overall SNV frequency for the regulatory proteins analyzed was 84.11% (3,764 out of 4,475 tumors). Further analysis revealed that missense mutations predominated as the SNP of interest. In terms of specific genes, SYNE1, KMT2C, APOB, SYNE2, AHNAK, ANK3, DST, HUWE1, LAMA2, and MACF1 stood out, with their mutation frequencies being 29%, 21%, 18%, 16%, 15%, 15%, 15%, 14%, 14%, and 13%, respectively (Figure 1D). Additionally, we noted an increase in the SNV frequency among these regulators in SKCM, UCEC, LUAD, BRCA, and LUSC.

To delve deeper into the function of RBP in tumor development and to identify factors or biological processes related to RBP, we evaluated the RBP potential index using the enrichment score (ES) from ssGSEA. We analyzed the variations in the RBP potential index, an established RBP marker, between tumor and normal tissues using TCGA pan-cancer data (Supplementary Figure S1C). Substantial discrepancies were observed in various cancers, including BRCA and LUSC, with an increased count of RBP found in most tumors, with KIRC being the exception. In particular, significant differences were observed in breast cancer (Figure 1E). To further scrutinize the factors contributing to these varied RBP potential index patterns, we studied the RBP potential index across different BRCA subtypes. The findings depicted in Figure 1F revealed that all luminal subtype patients had higher RBP potential indices compared to other patient types. This evidence suggests that RBPs have a significant role in pan-cancer, with their functions potentially differing among various molecular subtypes in breast cancer.

### Unraveling the role of CELF1 and its prognostic significance in luminal A breast cancer

**FIGURE 2:**
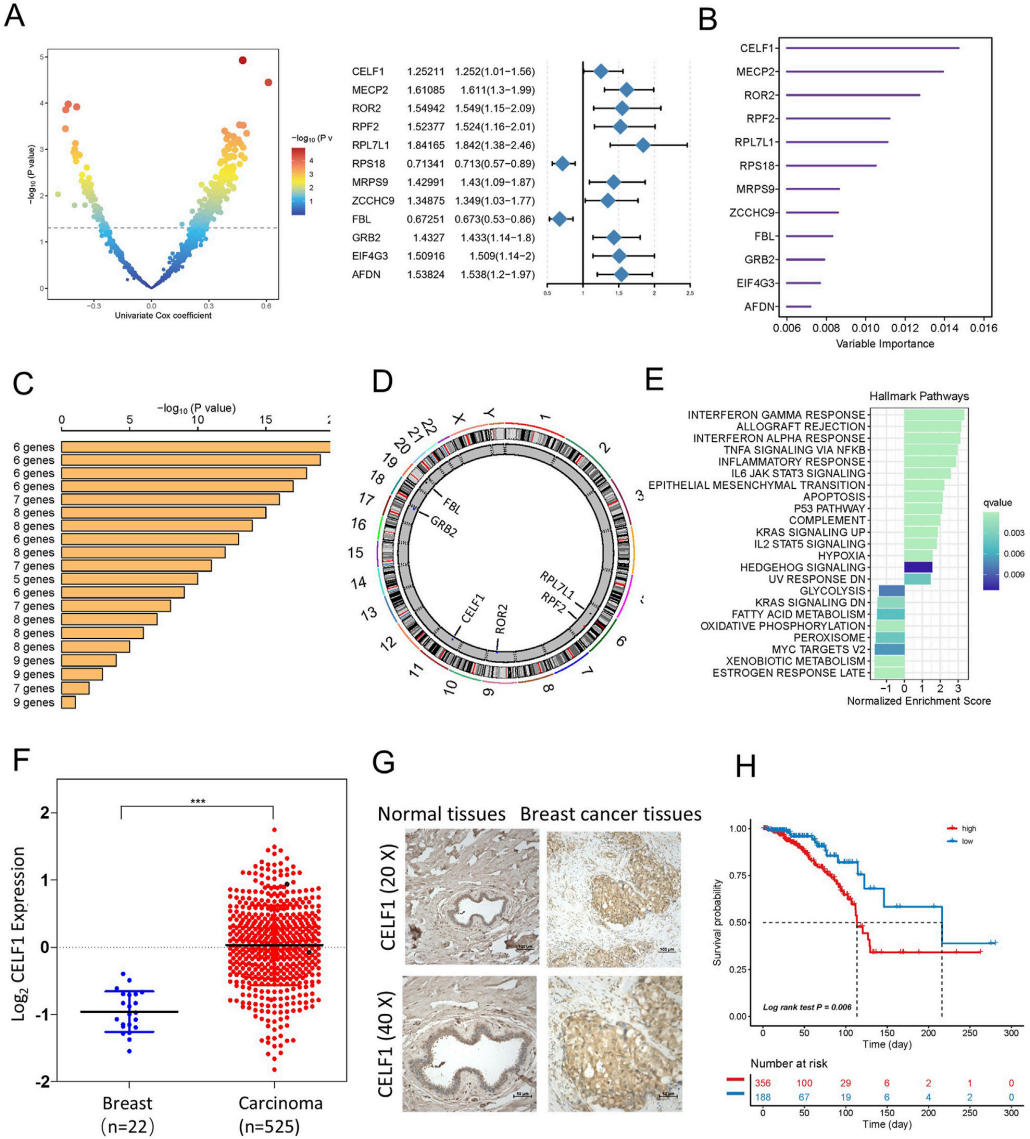
Identification of candidate RBP-responsive genes, along with CELF1 upregulation in breast cancer within a specific landscape of RBPs. **(A)** Left, volcano plot of univariable Cox analysis for RBPs in TCGA-BRCA [log2(HR) vs. −log10(P), BH adjusted]. Right, forest plot for the 12 top-ranked RBPs (HR with 95% CI; Wald test P). **(B)** A total of 12 genes were selected through random survival forest analysis. **(C)** Conducting Kaplan–Meier analysis on 2^12^–1 = 4,095 combinations, the top 20 signatures were organized according to their p-values. Among these signatures, six genes were selected for having a relatively large −log_10_ p-value and a small gene count. **(D)** The location of CNV alteration of six selected RBPs on 23 chromosomes using the TCGA-BRCA cohort. **(E)** Enrichment of Hallmark gene sets for genes that decrease in importance as CELF1 gene effect levels increase in a DepMap analysis. **(F)** Expression of CELF1 in TCGA-BRCA tumors vs. adjacent normal (UCSC Xena; overall comparison). The median and interquartile range are depicted by black lines in each group ***p < 0.001. **(G)** Immunohistochemical technique was employed to assess the expression levels of CELF1 in both breast carcinomas and adjacent noncancerous tissues. **(H)** Kaplan–Meier analysis on overall survival among luminal A breast cancer patients, categorizing samples into high- and low-CELF1 expression groups, with *p*-values derived from log-rank testing.

By employing an integrated analysis approach in luminal A subtype breast cancer, we identified RBPs with oncogenic capabilities. Our methodology involved retrieving gene expression data and clinical profiles of 545 eligible patients diagnosed with luminal A breast cancer from the TCGA database. Subsequent data processing revealed 1,337 RBPs extracted from this extensive patient cohort. To study the prognostic characteristics of RBP in luminal A subtype breast cancer patients, we applied univariate Cox regression analysis, and the outcomes indicated that 348 RBPs exhibited a significant association with the OS of patients diagnosed with luminal A breast cancer (p < 0.05, Supplementary Table S1, point above the dashed line in Figure 2A). Afterward, using the random forest supervised classification algorithm, we narrowed down the list of 348 OS-associated RBPs to 12 specific RBPs (CELF1, MECP2, ROR2, RPF2, RPL7L1, RPS18, MRPS9, ZCCHC9, FBL, GRB2, EIF4G3, and AFDN) (Figure 2B).

Since the combination of 12 genes could generate a total of 2^12^–1 = 4,095 permutations, we proceeded with additional Kaplan–Meier (KM) analysis to identify the most optimal permutation. By comparing the −log10 Plog‐rank values of these 4,095 permutations, we found that the six RBP permutations ranked highest. Given that an ideal permutation should contain minimal risk genes, we selected the ultimate prognostic signature consisting of six RBPs (CELF1, ROR2, RPF2, RPL7L1, FBL, and GRB2) (Figure 2C). The circular diagram depicts the chromosomal locations of CNV alterations in six RBPs (Figure 2D). Given CELF1’s top ranking, we additionally provide an overview of pan-cancer in Supplementary Figure S1D, integrating tumor mRNA expression, SNV, and CNA across 33 cancer types.

As our analysis above shows that CELF1 plays a vital role in lumina A breast cancer patients, we then proceeded to determine whether cancer cell lines with heightened CELF1 levels exhibit unique functionalities in contrast to cell lines with reduced CELF1 expression. As part of this evaluation, we first examined gene essentiality concerning CELF1 expression in cell lines using data from the DepMap database (Figure 2E). A direct relationship was observed between the levels of CELF1 and the significance of genes (signifying that elevated CELF1 levels correspond to reduced gene impact) implicated in late estrogen response, oxidative phosphorylation, and glycolysis. These particular genes display diminished importance in cancer cell lines included in the Cancer Cell Line Encyclopedia (CCLE) that exhibit overexpression of CELF1. This implies that cancer cell lines within the CCLE dataset, manifesting high CELF1 levels, possess an enhanced capacity to regulate metabolism across various cancer types.

To explore the function of CELF1 in human breast carcinomas, we conducted an examination of CELF1 gene expression data utilizing the TCGA database and found that the analyzed cohort comprised 545 cases of invasive ductal breast carcinomas and 22 samples of noncancerous breast tissues (Figure 2F) and identified a substantial upregulation of CELF1 expression in breast cancer in contrast to normal tissues. Moreover, the overabundance of CELF1 in breast carcinomas was validated through information obtained from the UALCAN database (Chandrashekar et al., 2017; Supplementary Figure S2A), which is in agreement with the immunohistochemistry staining results from human breast cancer tissue samples, providing support for the expression of the CELF1 protein (Figure 2G). We next determined whether CELF1 expression exhibits any correlation with the survival rates observed among breast cancer patients, by analyzing patients with luminal A subtype breast cancer in the TCGA database, stratified by CELF1 expression levels (Nagy et al., 2018; Figure 2H; Table 1). Patients were divided into two groups: those with high CELF1 expression and those with low expression. The Kaplan–Meier survival analysis reveals a marked difference in the median survival times between the two groups. In particular, the luminal A-type breast cancer patients with low CELF1 expression had a median survival time of 216.2 months. Conversely, those with high CELF1 expression had a significantly shorter median survival time of 113.5 months (*P* = 0.006). These findings underscore the potential impact of CELF1 expression on patient prognosis.

**TABLE 1:** Correlation between CELF1 expression and clinicopathological features.

Variable	Patient	CELF1		P-value
		Low	High	
Age (year)				
≤50	47	23	24	0.6788
>50	47	20	27	
No. of metastatic axillary nodes				
0	20	8	12	0.0301
1–3	45	10	35	
>3	29	15	14	
Diameter of the primary tumor				
≤30 mm	52	27	25	0.2951
>30 mm	42	20	22	
Histological grade				
1	18	9	9	0.4536
2	59	28	31	
3	17	11	6	
TNM staging				
I	39	9	30	0.0121
II	15	4	11	
III	24	14	10	
IV	16	9	7	

Abbreviation: TNM, tumor node metastasis.

### CELF1 is commonly overexpressed in the luminal subtype of luminal breast carcinomas and positively correlated with ESR1

**FIGURE 3:**
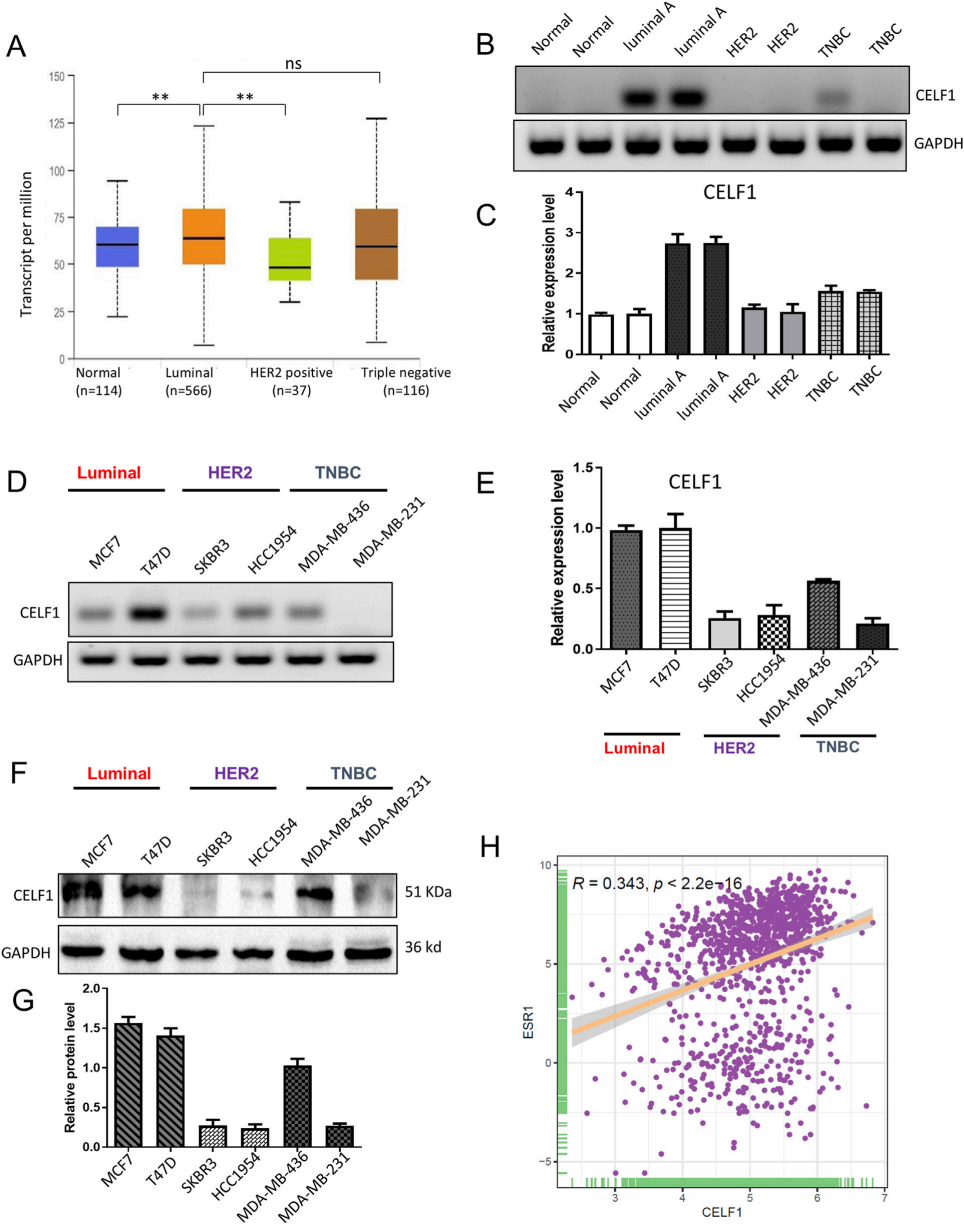
CELF1 is overexpressed in luminal A breast cancer and lowly expressed in triple-negative breast cancer cell line. **(A)** Subtype-stratified CELF1 expression based on TCGA-BRCA via UALCAN (luminal, HER2-enriched, and TNBC; subtype annotation available in the portal). Black lines in each group indicate a median with an interquartile range. **P < 0.01. ns, not significant. **(B)** Endpoint RT-PCR gels showing CELF1 and GAPDH in adjacent normal tissues (Normal) and tumor subtypes (Luminal A, HER2-enriched, and TNBC). Representative images are shown. **(C)** Quantitative real-time PCR (qRT-PCR) of CELF1 mRNA in the same tissue groups, normalized to GAPDH and expressed relative to the normal group. Data are shown as mean ± SEM from three independent experiments. **(D)** Endpoint RT-PCR gels for CELF1 and GAPDH in luminal cell lines (MCF7 and T47D), HER2 cell lines (SKBR3 and HCC1954), and TNBC cell lines (MDA-MB-436 and MDA-MB-231). Representative images are shown. **(E)** qRT-PCR measurement of CELF1 mRNA in the indicated cell lines, normalized to GAPDH and expressed relative to MCF7. Data are shown as mean ± SEM from three independent experiments. **(F,G)** Western blot analysis was performed on CELF1 protein levels within subtypes of luminal, HER2, and TNBC; GAPDH acted as the loading control **(F)** Protein expression levels were quantified relative to GAPDH using densitometric analyses **(G)** Data are expressed as means ± SEM. **(H)** The correlation between CELF1 and ESR1 expressions **P < 0.01. Every circle signifies a distinct human breast carcinoma sample. Correlation analysis was performed using Gene Expression Profiling Interactive Analysis (Tang et al., 2017).

The gene expression profiling of breast cancer allows for its classification into distinct subtypes. In our analysis of the UALCAN platform, we observed a significant overexpression of CELF1 in luminal breast cancer, while its expression was comparatively lower in the HER2-positive subtype (Figure 3A). To further investigate this, we compared CELF1 mRNA levels in different molecular subtypes of breast cancer tissues, including normal breast tissues, luminal, HER2+, and TNBC samples using RT-PCR. Our findings revealed that CELF1 exhibited the highest expression levels in luminal cells (Figures 3B,C; Supplementary Figure S2B). Additionally, when examining CELF1 expression in breast cancer cells at both transcriptional and protein levels, we observed a pronounced upregulation specifically in luminal breast cancer cells, as opposed to HER2-positive cells (Figures 3D–G; Supplementary Figure S2C). Given CELF1 overexpression in luminal A and the ER-positive nature of this subtype, we analyzed the CELF1–ESR1 correlation to determine whether CELF1 aligns with the ER-defined luminal transcriptional state. Our data reveal a significant positive association between CELF1 and ESR1 expressions (ERα) (Figure 3H; Supplementary Figure S3). Collectively, these results suggest that the role of CELF1 levels differs according to the specific molecular subtype of breast cancer.

### CELF1 regulates the proliferation, colony-forming ability, migration, and invasion of breast cancer cells

**FIGURE 4:**
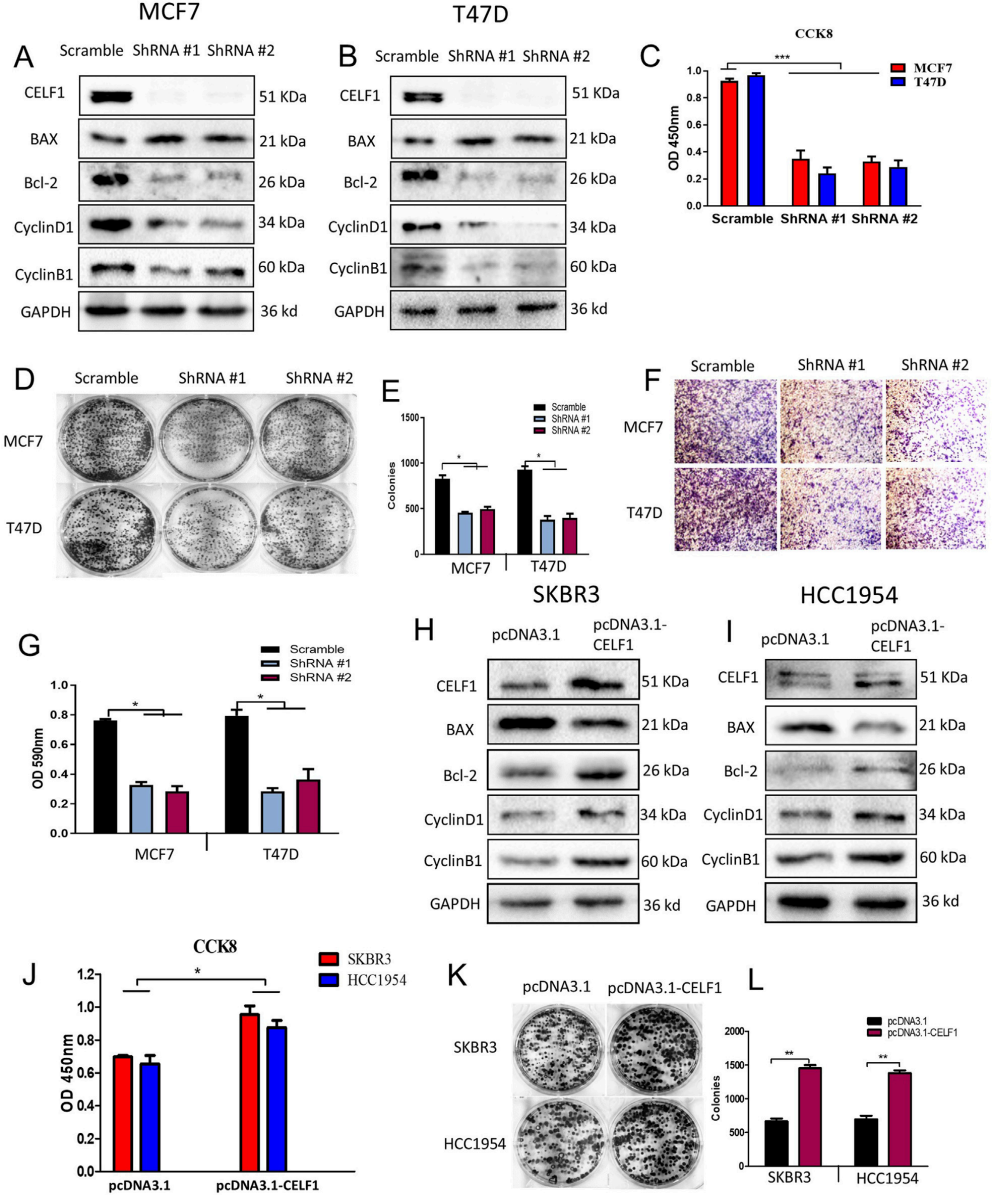
CELF1 regulates the proliferation, colony formation, migration, and invasion of breast cancer cells. **(A,B)** ShRNA-mediated silencing of CELF1, Bax, Bcl-2, cyclin D1, and cyclin B1 was analyzed using Western blotting in MCF7 cells **(A)** and T47D cells. **(B)** GAPDH acted as a loading control. For proteins CELF1, Bax, and Bcl-2, cropped lanes are obtained from the same gel (dividing lines between groups). Full uncropped blots are provided in Supplementary Figure file 2. **(C)** Knockdown of CELF1 in MCF7 and T47D cells; cell viability was assessed using CCK8 assays. Data are expressed as means ± SEM. ***P < 0.001. **(D,E)** Knockdown of CELF1 in MCF7 and T47D cells; cell growth was examined using plate assay **(D)** and the quantitative analysis **(E)**, at a density of 500 cells/well for 14 days. Data are expressed as means ± SEM. *P < 0.05. **(F,G)** Knockdown of CELF1 in MCF7 and T47D cells; cell invasion was examined using the transwell assay **(F)** and the quantitative analysis **(G)**. Data are expressed as means ± SEM. *P < 0.05. H, **(I)** Overexpression of CELF1 by transfection of the pcDNA3.1 vector and pcDNA3.1-CELF1 in SKBR3 **(H)** and HCC1954 cells **(I)**; CELF1, Bax, Bcl-2, cyclin D1, and cyclin B1 protein levels were analyzed using Western blotting; GAPDH was used as a loading control. **(J)** Overexpression of CELF1 in SKBR3 and HCC1954 cells; cell viability was assessed using CCK8 assays. Data are expressed as means ± SEM. *P < 0.05. **(K,L)** Overexpression of CELF1 in SKBR3 and HCC1954 cells; cell growth was examined using the plate assay **(K)**, and the quantitative analysis **(L)**, at a density of 500 cells/well for 14 days. Data are expressed as means ± SEM. **P < 0.01.

Subsequently, we reduced CELF1 levels in luminal breast cancer cells and enhanced CELF1 expression in HER2-positive breast cancer cells using shRNA and the pcDNA3.1-CELF1 plasmid, respectively. The functional role of CELF1 was assessed in breast cancer cells (Figure 4). The utilization of shRNA to hinder CELF1 gene expression in both MCF7 and T47D cell lines was validated through Western blot analysis, leading to a notable elevation in the BAX/Bcl2 ratio, while cyclin B1 and cyclin D1 levels were decreased following transfection with CELF-shRNA (Figures 4A,B). In contrast, when the pcDNA3.1-CELF1 plasmid was transfected into SKBR3 and HCC1954 cells, CELF1 expression increased, while the BAX/Bcl2 ratio decreased and the levels of cyclin B1 and cyclin D11 increased (Figures 4H,I). To explore the process of cellular proliferation, we evaluated cell viability using both the CCK8 assay and colony formation assay. Depletion of CELF1 significantly inhibited the growth of MCF7 and T47D cells compared to control cells (Figures 4C–E), whereas overexpression of CELF1 promoted cell growth in SKBR3 and HCC1954 cells (Figures 4J–L). Similarly, transwell assays revealed consistent patterns for invasion function (Figures 4F,G). Additionally, in the subsequent *in vivo* experiments of subcutaneous tumor implantation in nude mice, we also observed a substantial decrease in both tumor weight and volume in the CELF1-KO group. The expression of Ki-67, an indicator of cell proliferation, was also decreased. Collectively, these integrated findings demonstrate that CELF is associated with breast cancer aggressiveness.

### CELF1 promotes breast cancer cell aerobic glycolysis by regulating the expression of GLUT1

**FIGURE 5:**
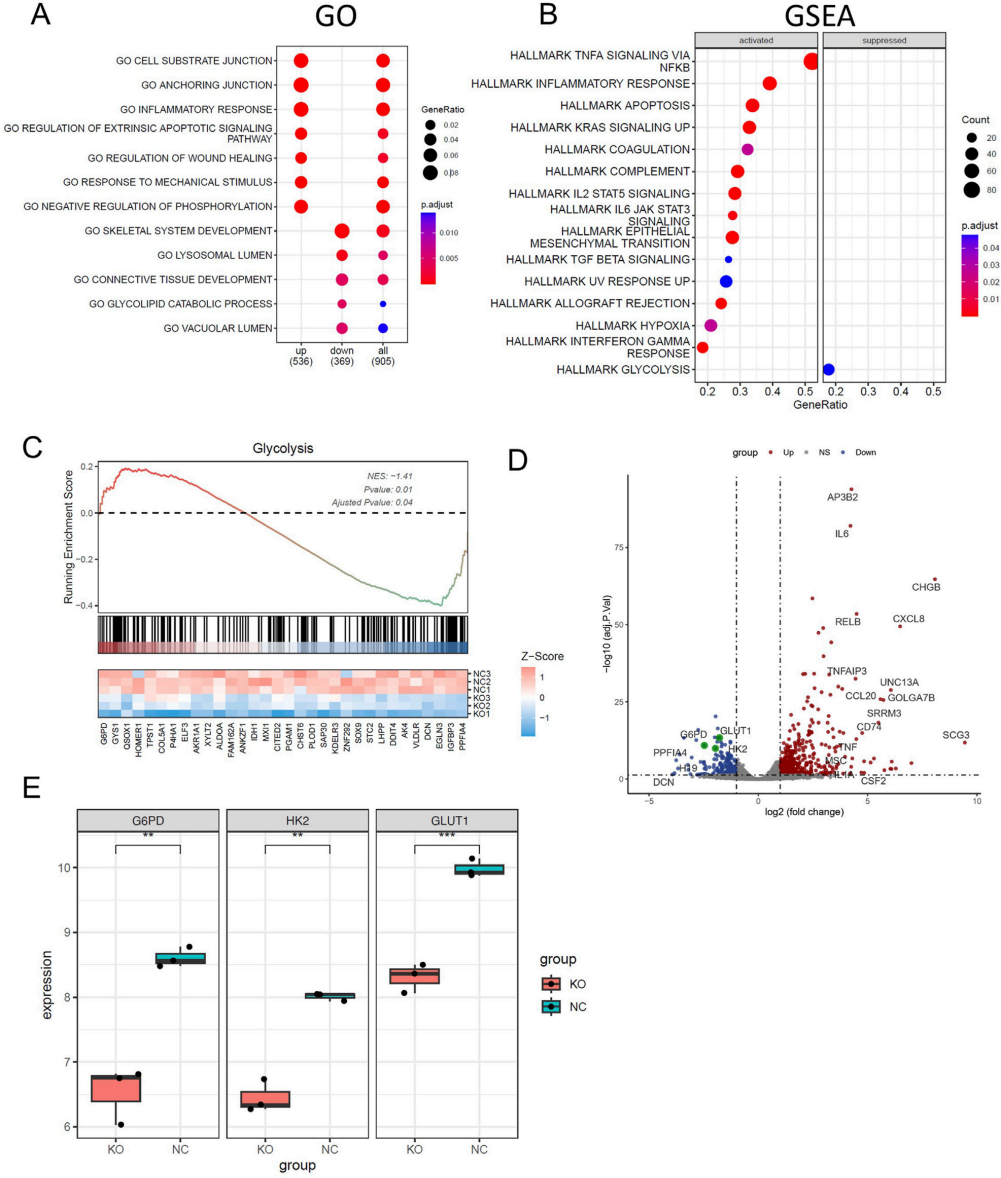
CELF1 promotes breast cancer cell aerobic glycolysis *in vitro*. **(A)** Enriched Gene Ontology (GO) terms of significantly differentially expressed genes between CELF1-knocked-out MCF7 cells and wild-type MCF7 cells. **(B)** GSEA shows the enriched hallmarks pathways between CELF1-knocked-out MCF7 cells and wild-type MCF7 cells. **(C)** GSEA shows the molecules that are significantly altered in the glycolytic pathway. **(D)** The volcano plot of differentially expressed genes between CELF1-knocked-out MCF7 cells and wild-type MCF7 cells. **(E)** Differential mRNA expressions of GLUT1, HK2, and G6PD in CELF1-knocked-out MCF7 cells and wild-type MCF7 cells.

For a more comprehensive understanding of how CELF1 functions in breast cancer, we performed RNA sequencing (RNA-seq) analysis on both CELF1-KO MCF7 cells and wild-type MCF7 cells (Figure 5). The differentially expressed genes (DEGs) were found to be significantly enriched in the glycolipid catabolic pathway (Figures 5A,B; Supplementary Figure S4C). The enrichment analysis of glycolipid metabolism in GSEA reveals that CELF1-KO cells exhibit inhibition of glycolytic-related genes and key enzymes (Figures 5C–E). This aligns with our observations in the Hallmark gene set enrichment analysis conducted in the CELF1 gene using the DepMap platform, which underscores the glycolysis pathway as prominently impacted (Figure 2E).

**FIGURE 6:**
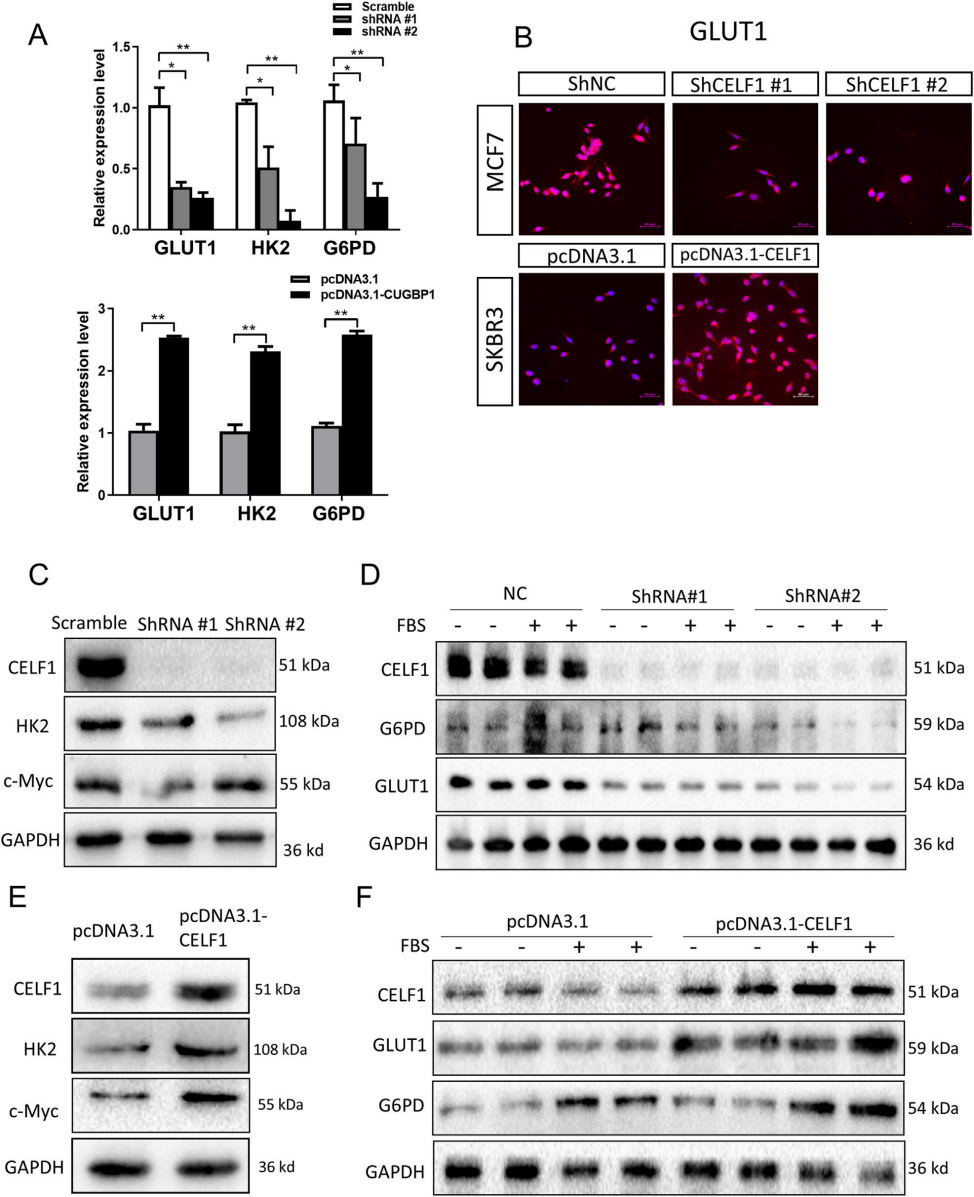
CELF1 governs aerobic glycolysis by regulating GLUT1 levels. **(A)** Relative RNA expressions of GLUT1 HK and G6PD in CELF1-KO MCF7 cells (upper) and CELF1-overexpressed SKBR3 cells (lower) measured using real-time PCR. Data are expressed as means ± SEM. *P < 0.05.**P < 0.01. ***P < 0.001. **(B)** Immunofluorescence of GLUT1 in CELF1-KO MCF7 cells and CELF1-overexpressed SKBR3 cells. Scale bar: 50 μm. **(C,D)** Protein levels of CELF1, HK, c-Myc, G6PD, and GLUT1 in MCF7 cells after the transfection of control and CELF1 shRNA were determined using Western blotting. Cell lysates were collected with or without pretreated 20% fetal bovine serum (FBS) for 4 h. GAPDH served as an internal control. **(E,F)**. Protein levels of CELF1, HK, c-Myc, G6PD, and GLUT1 in SKBR3 cells after the transfection of the pcDNA3.1 and pcDNA3.1–CELF1 vectors were determined using Western blotting. Cell lysates were collected with or without pretreated 20% FBS for 4 h. GAPDH served as an internal control.

**FIGURE 7:**
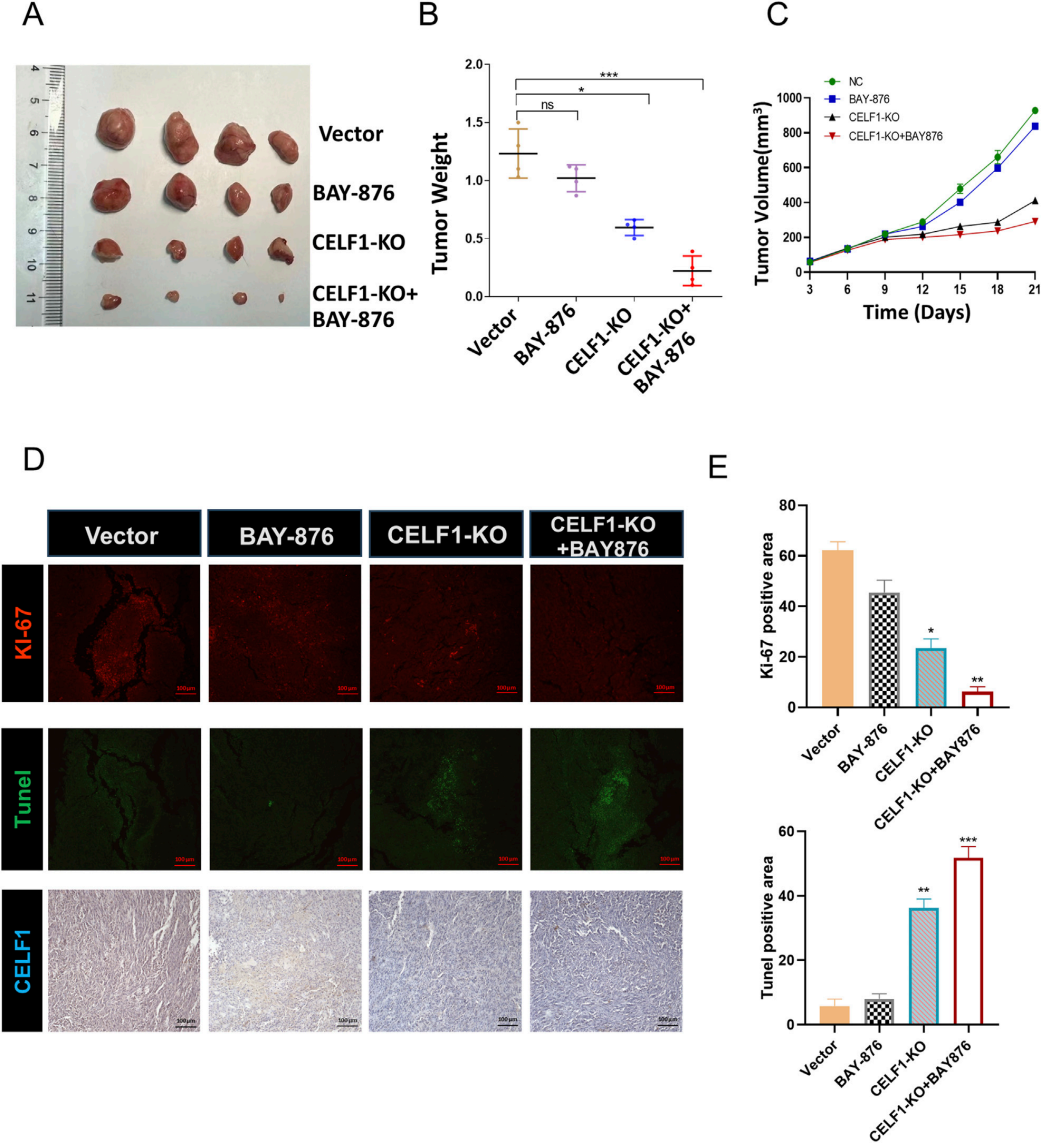
CELF1-KO combined with the GLUT1 inhibitor BAY876 inhibits the proliferation of breast cancer cells *in vivo*. **(A)** Nude mice were injected with CELF1-KO T47D cells to establish subcutaneous tumor models. The mice were orally administered BAY-876 for 3 consecutive days (once daily). The outcomes are visually represented as the images of the subcutaneous tumor tissues **(A)**, tumor weights **(B)**, and tumor volumes **(C)**. F (3,12) = 40.89, *P < 0.05 and ***P < 0.001. **(D,E)**. Immunofluorescence and IHC of Ki67, Tunel, and CELF1 in the control group, BAY-876 group, CELF1-KO group, and CELF1-KO combined with the BAY-876 group. Scale bars = 100 *μ*m. **(E)** Statistical analysis of the data shown in the upper panel yielded F (3,12) = 188.7, and for the lower panel F (3,12) = 285.0 (*P < 0.05.**P < 0.01. ***P < 0.001).

**FIGURE 8:**
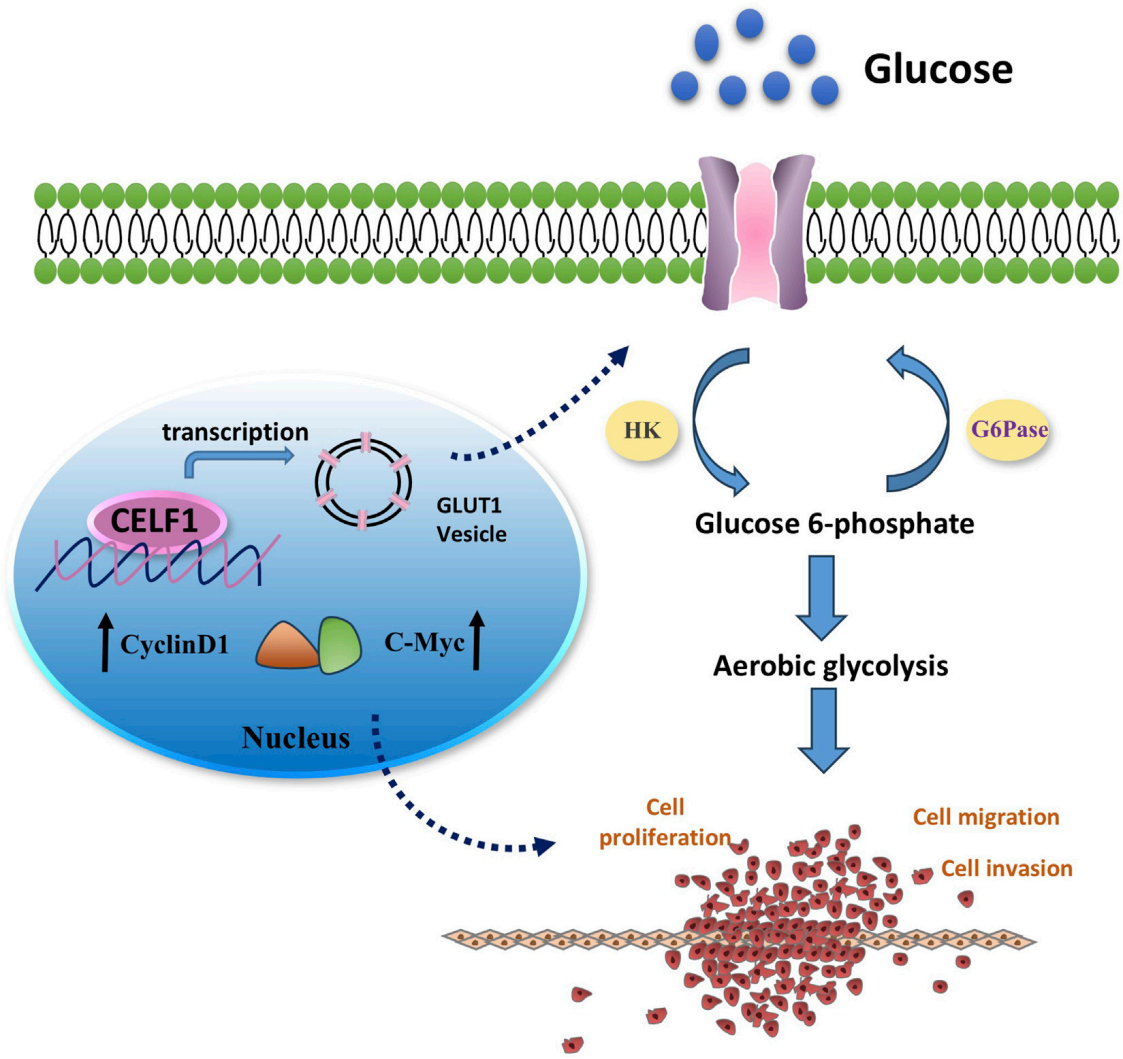
Mechanism diagram illustrating the involvement of CELF1 in aerobic glycolysis in breast cancer. It is well known that many aggressive tumors develop dysregulated metabolism; the glycolytic pathway was closely correlated to the vitality of tumors. Our transcriptomic analysis results suggest that CELF1 alterations impact the glycolysis process, and GLUT1 is the main molecule among all the volatile metabolites. Therefore, we focused on the genes related to aerobic glycolysis. As shown in Figure 8, the expression of GLUT1 is substantially decreased in the CELF1-knocked-out group, and so is the expression of key enzymes HK and G6PD in the aerobic glycolysis process (Figure 8). In addition, knockout of CELF1 affects the expression of cyclin D1 and *c-Myc*, suggesting that the occurrence, invasion, and metastasis processed are changed accordingly in the tumor tissues.

It is well known that many aggressive tumors develop dysregulated metabolism; the glycolytic pathway was closely correlated to the vitality of tumors. Our transcriptome results suggest that the mRNA expressions of G6PD and GLUT1 changed in RNA-Seq analysis. Therefore, we focused on the genes related to aerobic glycolysis. As shown in Figure 6, the expression of GLUT1 is substantially decreased in the CELF1 knock-out group, and so are the expressions of key enzymes HK and G6PD in the aerobic glycolysis process (Figures 6C,D). Conversely, the CELF1 overexpression group led to an upregulation of the expressions of GLUT1, HK, and G6PD (Figures 6B,E,F). The alterations in CELF1 expression also impact the expressions of cyclin D1 and c-Myc, thereby implying a consequential modification in the occurrence, invasion, and metastasis processes within tumor tissues. The tumor weight and volume of mice in the CELF1-KO group decreased significantly compared with those in the control group, and the expression of the cell proliferation indicator Ki-67 also decreased; we also found that CELF1-KO combined with the GLUT1 inhibitor BAY876 inhibits the proliferation of breast cancer cells *in vivo* in the subcutaneous tumor implantation experiment in nude mice (Figures 7A–E). As schematically described in Figure 8, CELF1 promotes breast cancer cell aerobic glycolysis by regulating the expression of GLUT1.

## Discussion

Recent comprehensive genomic studies have revealed a diverse array of RBPs that experience frequent mutations and gene amplifications across various types of tumors (Sebestyén et al., 2016; Kechavarzi and Janga, 2014). In this study, we have investigated how RBPs may function across various types of cancers, with a specific focus on breast cancer receptor-positive molecular subtype—luminal A. Using comprehensive analysis across TCGA, we found that RBPs show high frequencies of copy number amplification in most cancer types, and they also exhibit a wide range of non-silent mutations. We subsequently revealed the importance of RNA-binding protein CELF1 in luminal A subtype breast cancer. Unlike MSI proteins that sustain cancer stemness in triple-negative breast cancer or HuR’s regulation of Snail/MMP9-driven metastasis in HER2+ tumors (Chen et al., 2022), CELF1 uniquely drives metabolic reprogramming in luminal A. This aligns with its role in reshaping mitochondrial metabolism in diabetic cardiomyopathy (Belanger et al., 2018), suggesting tissue-agnostic metabolic functions.

Several studies have revealed that CELF1 demonstrates frequent upregulation in glioma, oral squamous-cell carcinomas, and hepatocellular carcinoma (Xia et al., 2015; Talwar et al., 2013; Kim et al., 2017), and its oncogenic properties make it a crucial target for influencing cell proliferation and growth and precisely regulating the cell cycle. Contrary to G. David et al.'s report that CELF1 mRNA alone lacked a prognostic value (Géraldine et al., 2018), our IHC-based analysis of luminal A specimens revealed that CELF1 protein overexpression strongly correlates with poor survival. This discrepancy likely stems from the following: (i) methodological differences (subtype-stratified IHC vs. pan-cancer mRNA sequencing) and (ii) post-transcriptional regulation by ELAVL1, which stabilizes CELF1 protein in aggressive subtypes, as observed in oral SCCs (House et al., 2015).

Emerging evidence suggests that CELF1 dysregulation can influence crucial molecular processes involved in cancer pathogenesis. This study demonstrates that CELF1 overexpression contributes to a downregulation in the BAX/Bcl2 ratio and upregulation in cyclin B1 and cyclin D1 levels, thereby promoting cell viability, colony formation, and invasion. Conversely, the downregulation of CELF1 expression has the opposite effect. A similar phenomenon was detected in HER2-positive breast cancer, in which CELF1 activates the translation of C/EBPb-LIP isoform and further promotes the proliferation of cancer cells (Arnal-Estapé et al., 2010). Aberrant splicing events of CELF1 can lead to the production of abnormal isoforms, contributing to the translational activation of genes that drive epithelial–mesenchymal transition (EMT) and, consequently, tumor progression (Chaudhury et al., 2016). Diverging from preceding investigations, our research employed a varied array of molecular subtypes of cell lines and breast cancer tissues, leading us to discern the impact of CELF1 on apoptosis and cell cycling progress. Our findings confirmed that CELF1 promotes the aggressiveness of breast cancer cells, which was supported by both *in vitro* and *in vivo* experiments. Moreover, our findings indicated a direct relationship between CELF1 expression and the existence of ESR1 (ERα), a marker for the luminal subtype. These results further emphasize the importance of molecular subtype-specific studies in understanding the complexity of cancer biology.

Metabolic dysregulation represents a pivotal attribute within the intricacies of tumorigenesis. CELF1 can regulate alternative splicing patterns in various genes associated with cancer. Currently, few researchers are elucidating the intricate relationship between CELF1 and metabolic processes. Our previous study revealed that CELF1 exerts its influence by modulating the LIP/LAP molar ratio, thereby controlling the diverse mRNA splicing profiles of INSR in breast cancer cells and affecting cell aggressiveness (Huang et al., 2020). This intriguing observation engenders novel inquiries. Considering the significance of the insulin receptor (INSR) as a gene intricately linked to metabolic processes, it stimulates our curiosity as to whether CELF1’s regulatory control over INSR splicing also exerts an influence on metabolic functions. Our discovery that CELF1 upregulates GLUT1 extends prior work on endocrine resistance. Notably, ER+ tumors with TCA cycle disruption (e.g., via MAT1A downregulation) exhibit similar metabolic vulnerabilities (Santaliz-Casiano et al., 2023; Brechbuhl et al., 2024). This suggests that CELF1-driven glycolysis may synergize with ESR1-mutant-induced OXPHOS suppression—a mechanism recently implicated in fulvestrant resistance (Brechbuhl et al., 2024; Saatci et al., 2021).

In conclusion, our study has provided valuable insights into the complex roles of RBPs in tumorigenesis, with an emphasis on their subtype-specific impacts on breast cancer. We highlighted CELF1 as a potentially key regulator of metabolic reprogramming in luminal A breast cancer, thereby shedding light on new directions for future research and treatment strategies. However, there are significant unresolved aspects that warrant further research to gain a comprehensive understanding. First, the reliance on TCGA data and cell line models may not fully recapitulate the heterogeneity of human breast tumors, particularly in accounting for microenvironmental influences such as stromal interactions and immune cell infiltration. Second, although we identified CELF1’s role in glycolytic reprogramming via GLUT1, the precise molecular mechanisms linking CELF1 to ESR1 signaling remain unresolved. Therefore, further exploration is warranted to elucidate the interplay between CELF1 and ER in breast cancer. It remains to be investigated whether ER is involved in the glycolytic metabolism process and whether CELF1 influences endocrine resistance in ER-positive breast cancer.

## Novelty and impact

The specific impact of RBPs on the development of breast cancer remains uncertain. Our research has led us to uncover a set of genes enriched in breast cancer influenced by CELF1, which shows distinctive prognostic value specifically in luminal A breast cancer. It is well known that the dysregulated metabolism of glucose and lipids is strongly associated with cancer incidence and aggressiveness. So far, there are a limited number of studies exploring the relationship between CELF1 and metabolism. Our data suggest that CELF1 is expressed and functional in different molecular subtypes of breast cancer; additionally, CELF1 drives the metabolic preference for aerobic glycolysis in breast carcinomas by modulating the expression of GLUT1.

## Data Availability

The gene expression data are available from the cBioportal database, https://www.oncomine.org/, GEPIA (Gene Expression Profiling Interactive Analysis).

## Glossary

CELF1: CUGBP Elav-like family member 1
ER: Estrogen receptor
TCGA: The Cancer Genome Atlas
IHC: Immunohistochemistry
GLUT1: Glucose transporter 1
G6PD: Glucose-6-phosphate dehydrogenase
HK2: Hexokinase 2
ACC: Adrenocortical carcinoma
BLCA: Bladder urothelial carcinoma
BRCA: Breast invasive carcinoma
CESC: Cervical squamous cell carcinoma and endocervical adenocarcinoma
CHOL: Cholangiocarcinoma
COAD: Colon adenocarcinoma
DLBC: Lymphoid neoplasm diffuse large B-cell lymphoma
ESCA: Esophageal carcinoma
GBM: Glioblastoma multiforme
HNSC: Head and neck squamous cell carcinoma
KICH: Kidney chromophobe
KIRC: Kidney renal clear cell carcinoma
KIRP: Kidney renal papillary cell carcinoma
LAML: Acute myeloid leukemia
LGG: Brain lower-grade glioma
LIHC: Liver hepatocellular carcinoma
LUAD: Lung adenocarcinoma
LUSC: Lung squamous cell carcinoma
MESO: Mesothelioma
OV: Ovarian serous cystadenocarcinoma
PAAD: Pancreatic adenocarcinoma
PCPG: Pheochromocytoma and paraganglioma
PRAD: Prostate adenocarcinoma
READ: Rectum adenocarcinoma
SARC: Sarcoma
SKCM: Skin cutaneous melanoma
STAD: Stomach adenocarcinoma
TGCT: Testicular germ cell tumor
THCA: Thyroid carcinoma
THYM: Thymoma
UCEC: Uterine corpus endometrial carcinoma
UCS: Uterine carcinosarcoma
UVM: Uveal melanoma
