# Supplementary material for: CELF1 promotes aerobic glycolysis and an aggressive phenotype in ER-positive breast cancer via GLUT1 regulation
Source: Front Genet. 2025 Nov 12;16:1687066. doi: 10.3389/fgene.2025.1687066 (PMC12646540; doi:10.3389/fgene.2025.1687066)
Supplement: Supplementary file 1 [file Presentation1.pdf]

Supplementary Material

1 Supplementary Figures and Tables

1.1 Supplementary Figures

FigS.1

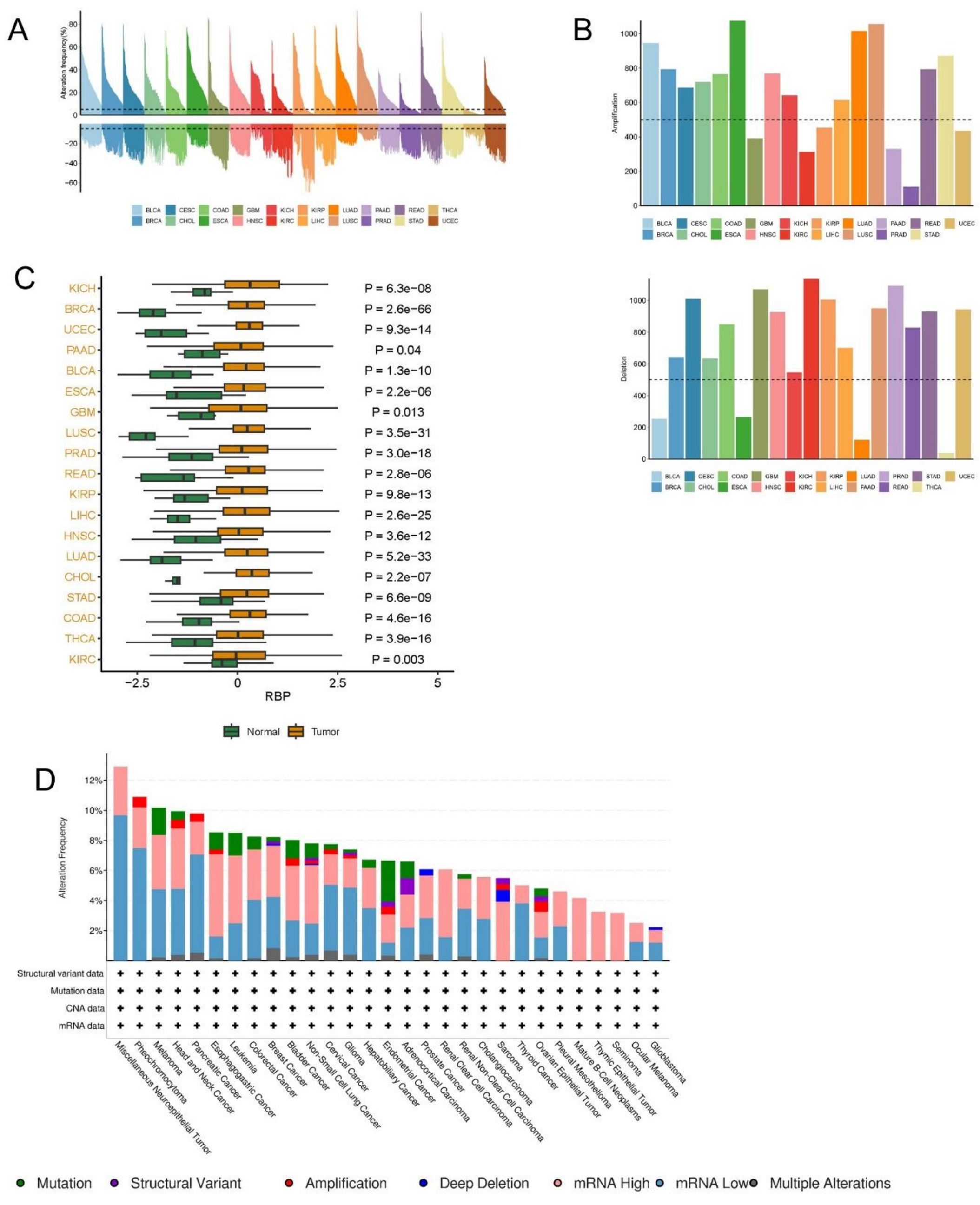

Supplementary Figure 1. Pan-cancer landscape of RBPs and a TCGA PanCancer Atlas overview of CELF1.
A. Histogram shows the frequency of somatic copy number alterations (deletions or amplifications) for all known RBPs in each cancer type.
B. Amplified RBPs (segment mean > 0.2) and deleted RBPs (segment mean < −0.2) are shown separately.
C. The different RBP census between tumor and normal tissues among cancers.
D. TCGA PanCancer Atlas overview of CELF1 across 33 cancer types, summarizing tumor mRNA expression, SNVs, and CNAs.

FigS.2

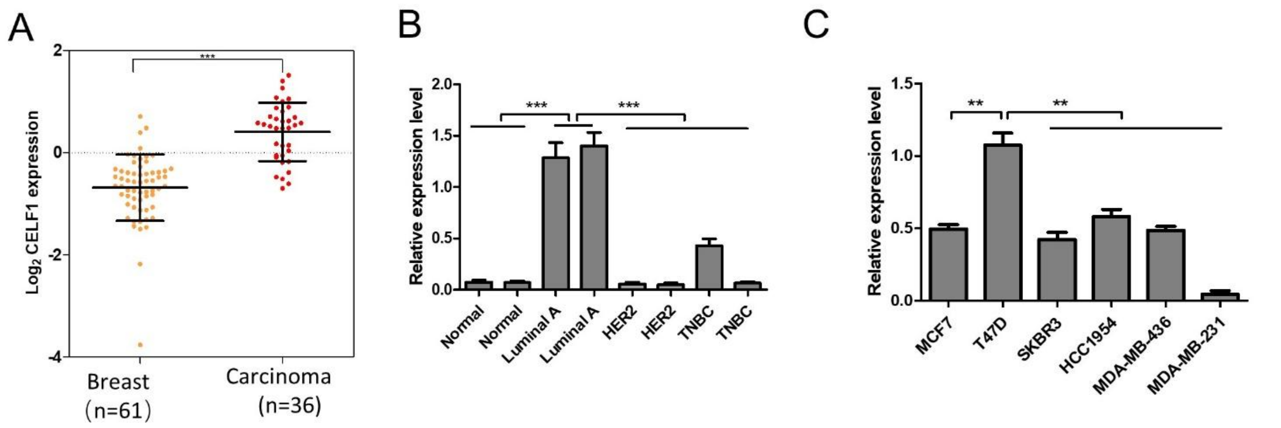

Supplementary Figure 2. Expression of CELF1 in molecular subtypes of breast cancer.
A. Expression of CELF1 correlates with the pathological grade of breast carcinomas in the UALCAN analysis website (http://ualcan.path.uab.edu/index.html) based on individual cancer stages. Black lines in each group indicate median with interquartile range. *P<0.05. ns, denotes not significant.
B. Differential molecular subtypes of breast cancer tissues were collected following RNA extraction and CELF1 relative RNA expression measured by qPCR. Data are means ±SEM. ***P<0.001， **P<0.01.
C. Differential molecular subtypes of breast cancer cell lines were collected following RNA extraction and CELF1 relative RNA expression measured by qPCR. Data are means ±SEM. **P<0.01.

FigS.3

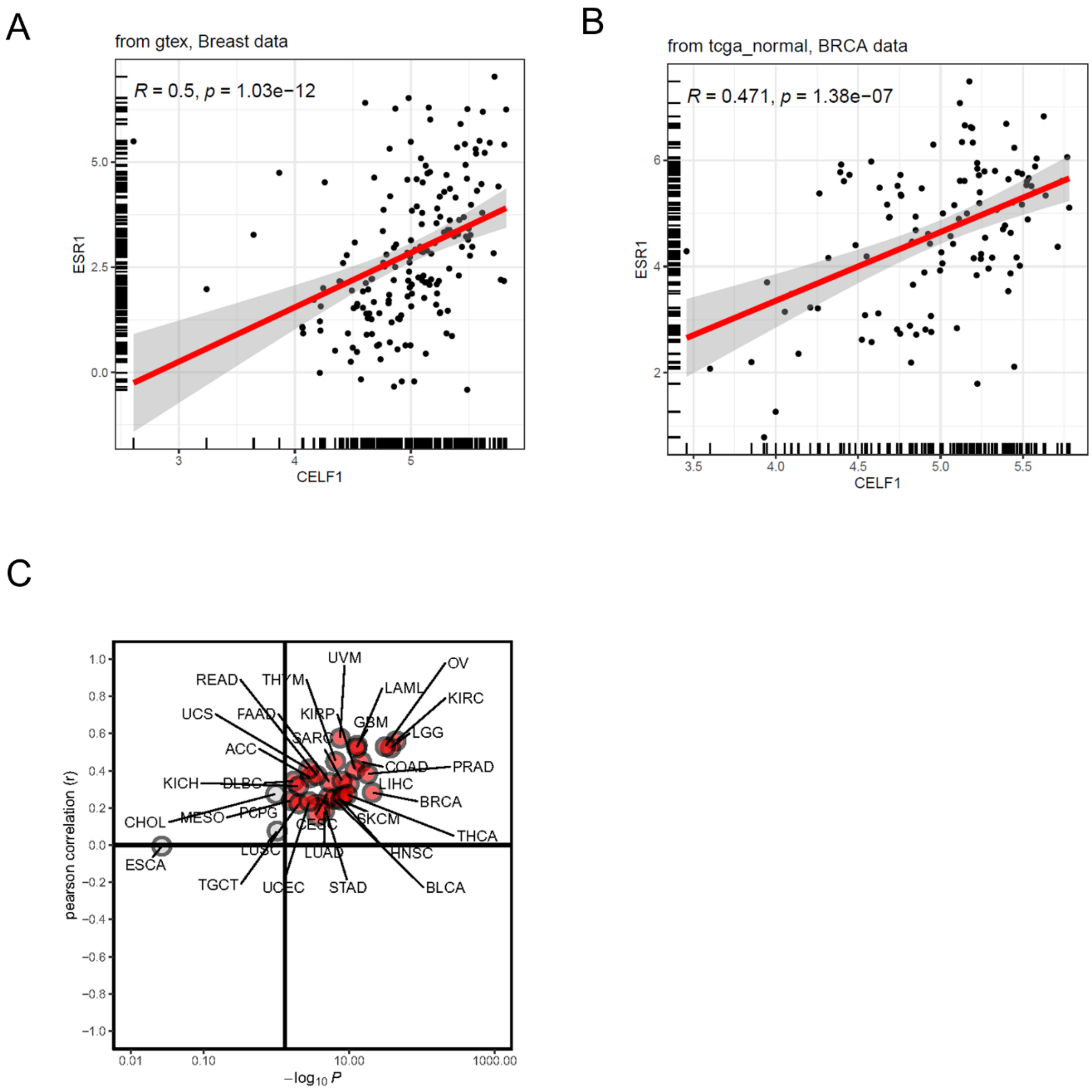

Supplementary Figure 3. The Relationship between CELF1 and ESR1 in normal breast tissue and adjacent cancer tissue.

A. The correlation between CELF1 and ESR1 expression in GTEx normal breast tissue. P=1.03e-12. Each circle represents an individual sample of human normal breast tissue.

B. The correlation between CELF1 and ESR1 expression in adjacent cancer tissue. P=1.38e-07. Each circle represents an individual sample of TCGA human adjacent breast cancer tissue.

C. The relationship between CELF1 and individual ESR1 expression in cancer samples was examined using data obtained from the Cancer Genome Atlas (TCGA). Each dot on the graph represents a specific tissue type or cancer type.

FigS.4

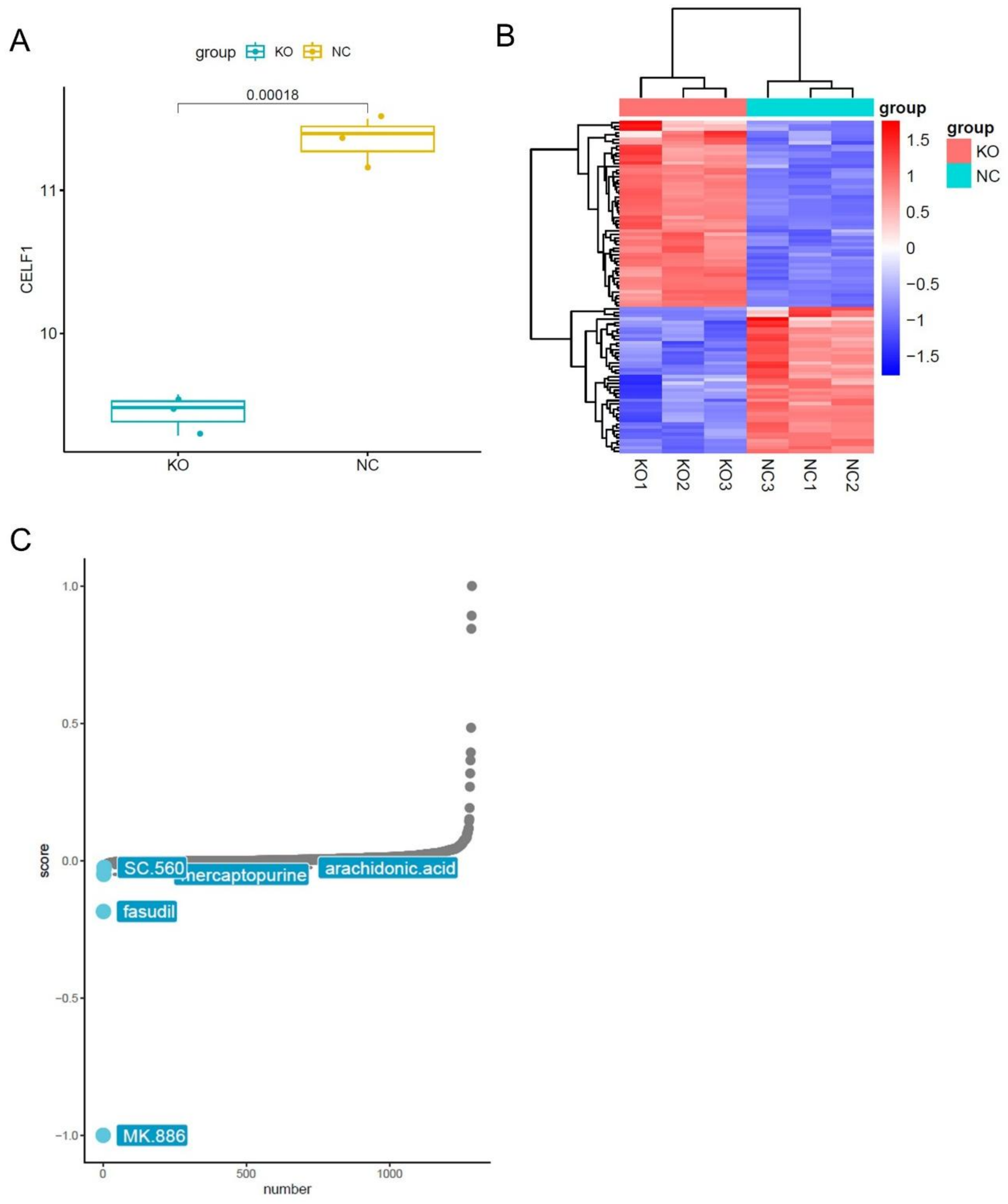

Supplementary Figure 4. Transcriptomics analysis of CELF1 knock-out in MCF7 cells.
A. The mRNA expression levels of CELF1 in CELF1 knock-out MCF7 cells and wild-type MCF7 cells.
B. Hierarchical clustering and heatmap of significantly differentially expressed genes between CELF1 knock-out and control MCF7 cells.

C. The top 5 compounds with the highest reversal potency were depicted in the panel, based on the calculation of CMap scores for differentially expressed genes using CELF1 knock-out MCF7 cells and wild-type MCF7 cells as the query signature.

1.2 Supplementary Tables

Table 1 Univariate cox regression for RBP filtering in luminal A breast cancer

	gene	HR	z	pvalue	lower	upper
19	RPS18	0.713409	-3.02701	0.00247	0.573293	0.887771
20	RPS11	0.666449	-3.25202	0.001146	0.521859	0.851101
27	RPS27	0.680957	-3.22233	0.001272	0.539033	0.860248
33	HSP90AB1	1.378816	2.519931	0.011738	1.07399	1.770159
37	RPS4X	0.753939	-2.2724	0.023062	0.590932	0.96191
46	RPL13A	0.704238	-2.71979	0.006532	0.546992	0.906687
47	RPL3	0.792212	-1.99772	0.045747	0.630369	0.995607
53	RPS2	0.740533	-2.38742	0.016967	0.57869	0.947638
59	RPLP2	0.647582	-3.87764	0.000105	0.519892	0.806634
60	RPLP0	0.766368	-2.33861	0.019356	0.613176	0.957832
65	RPS8	0.715249	-2.75468	0.005875	0.563512	0.907845
68	RPS21	0.69488	-2.82297	0.004758	0.539695	0.894685
80	RPS25	0.758013	-2.28233	0.02247	0.597515	0.961623
81	RPL12	0.765943	-2.18022	0.029241	0.602687	0.973422
83	RPL11	0.687156	-3.21021	0.001326	0.546476	0.864052

92	RPL29	0.754884	-2.38283	0.01718	0.599007	0.951323
98	RPL27	0.787533	-2.02451	0.042918	0.624949	0.992414
99	RPL35	0.784378	-1.98104	0.047587	0.616841	0.997419
101	RPL10	0.763224	-2.25795	0.023949	0.603657	0.964969
102	RPL4	0.748391	-2.37221	0.017682	0.589021	0.95088
103	RPL5	0.771248	-2.00845	0.044595	0.598565	0.993749
109	RPS16	0.676963	-3.84402	0.000121	0.554848	0.825953
113	RPL32	0.744806	-2.40313	0.016255	0.58571	0.947116
116	RPS19	0.731285	-2.63689	0.008367	0.579514	0.922804
122	RPS3	0.741496	-2.19094	0.028456	0.567428	0.968961
123	RPL13	0.74497	-2.29959	0.021471	0.579644	0.95745
128	PTMA	0.771094	-2.14938	0.031604	0.608364	0.977352
131	RPL24	0.696301	-2.91652	0.00354	0.545951	0.888057
141	RPS13	0.675005	-3.12096	0.001803	0.527365	0.863977
148	RPL36	0.736717	-2.56784	0.010233	0.583465	0.930222
149	RPL18	0.718705	-2.6309	0.008516	0.561933	0.919215
160	TPT1	0.745438	-2.51271	0.011981	0.592776	0.937418
179	RPS9	0.729513	-2.7603	0.005775	0.583148	0.912614
184	RPS24	0.776754	-2.0699	0.038462	0.611497	0.986672

187	RPSA	0.672478	-3.37451	0.000739	0.534061	0.846769
188	S100A16	0.787974	-2.04727	0.040631	0.627244	0.989889
198	RPS14	0.744365	-2.51145	0.012024	0.591189	0.937229
212	EZR	1.444796	2.811487	0.004931	1.117895	1.867291
220	RPL31	0.730302	-2.54082	0.011059	0.573073	0.930669
229	FAU	0.721811	-2.86687	0.004146	0.577608	0.902015
231	EIF4G2	1.309286	2.047598	0.040599	1.0116	1.694573
232	RPS15	0.716808	-2.8073	0.004996	0.568133	0.90439
236	RPS20	0.706382	-2.63348	0.008452	0.545365	0.914938
286	RPL28	0.772457	-2.17602	0.029554	0.612183	0.974691
290	RPS28	0.713976	-2.63938	0.008306	0.555943	0.91693
323	RPL27A	0.639385	-3.80991	0.000139	0.507971	0.804795
329	RPL18A	0.705341	-2.77873	0.005457	0.551402	0.902257
337	RPL38	0.719847	-2.5589	0.0105	0.559622	0.925946
356	SNRPD2	0.703308	-2.72963	0.00634	0.54625	0.905524
360	LGALS3	0.787302	-1.96236	0.04972	0.620025	0.999707
392	RPS7	0.726161	-2.68673	0.007215	0.574985	0.917084
414	RPL37	0.774825	-2.10919	0.034928	0.611288	0.982112
439	YWHAG	1.326818	2.024323	0.042937	1.009031	1.744691
489	FBL	0.672507	-3.18797	0.001433	0.526944	0.858279

511	RPL14	0.670078	-3.29884	0.000971	0.528226	0.850023
529	PPIB	0.765168	-2.22211	0.026276	0.604264	0.968917
536	EIF3H	1.388448	2.423538	0.01537	1.064788	1.810491
541	DDX17	1.343331	2.106107	0.035195	1.020692	1.767955
566	YWHAB	1.615359	3.613601	0.000302	1.245395	2.095227
587	SRRM2	1.404906	2.362803	0.018137	1.059675	1.862611
604	EIF4A2	1.295955	2.005617	0.044897	1.005919	1.669618
613	EIF4G1	1.311983	2.18996	0.028527	1.028928	1.672906
614	ARCN1	1.337043	2.064974	0.038925	1.01488	1.761472
659	GRB2	1.4327	3.091417	0.001992	1.14065	1.799525
696	STAU1	1.275844	2.015998	0.0438	1.006794	1.616794
719	RBM3	0.768185	-2.01072	0.044355	0.59405	0.993365
751	SBDS	1.461358	2.570413	0.010158	1.094279	1.951573
825	STIP1	1.35306	2.424177	0.015343	1.059611	1.727777
839	MRPS35	1.251504	2.085468	0.037027	1.013593	1.545257
874	PCNP	1.332353	2.166408	0.03028	1.027721	1.727281
888	PRRC2A	1.305572	1.97086	0.04874	1.001475	1.702008
906	HNRNPDL	1.347168	2.204021	0.027523	1.033549	1.755951
929	XRN2	1.373488	2.441836	0.014613	1.064629	1.771949

946	MAT2A	1.626548	3.163433	0.001559	1.203296	2.198675
976	FAM120A	1.319184	2.004508	0.045016	1.006175	1.729567
993	TCP1	1.533442	3.01436	0.002575	1.161301	2.024836
1013	NONO	1.281153	2.042738	0.041078	1.01009	1.624957
1035	HSPA9	1.344108	2.300648	0.021412	1.044765	1.729217
1054	HTATSF1	1.405817	2.646903	0.008123	1.092424	1.809116
1098	POLR2A	1.315194	1.967975	0.049071	1.001116	1.727808
1127	CPNE3	1.437806	2.844707	0.004445	1.119559	1.846519
1141	GOT2	1.361786	2.411031	0.015907	1.059473	1.750362
1171	SRSF6	1.383971	2.359993	0.018275	1.056627	1.812727
1269	BRD2	1.445695	2.674633	0.007481	1.103501	1.894003
1283	MTDH	1.402662	2.770455	0.005598	1.104055	1.782032
1291	SEC31A	1.430127	2.47987	0.013143	1.07789	1.897469
1340	LARP1	1.379405	2.533537	0.011292	1.075536	1.769126
1358	SLBP	1.298974	2.075896	0.037904	1.014715	1.662864
1366	PTPN1	1.288039	2.20156	0.027696	1.028167	1.613595
1401	PUM2	1.355626	2.302491	0.021308	1.046303	1.756394
1458	NAA50	1.297532	2.08288	0.037262	1.015489	1.657908
1487	USO1	1.405045	2.509138	0.012103	1.077271	1.832549
1504	ZC3H15	1.416948	2.386133	0.017027	1.064222	1.886582

1507	KPNB1	1.353019	2.355361	0.018505	1.052064	1.740066
1530	UBQLN1	1.525585	2.984654	0.002839	1.156052	2.013241
1547	NDRG1	1.467632	3.12465	0.00178	1.153732	1.866935
1556	DDX3X	1.42713	2.612388	0.008991	1.092889	1.863592
1561	SRP72	1.363409	2.475444	0.013307	1.06668	1.742681
1597	PAICS	1.445625	2.666345	0.007668	1.102561	1.895435
1678	SON	1.377758	2.404181	0.016209	1.060999	1.789087
1728	PRKDC	1.294604	2.130232	0.033152	1.020853	1.641765
1746	HUWE1	1.459275	2.970444	0.002974	1.137197	1.872572
1782	BAZ1B	1.326781	2.392921	0.016715	1.052491	1.672553
1902	PAIP1	1.294386	2.029391	0.042419	1.008867	1.660711
1918	MRPS15	0.755018	-2.18972	0.028545	0.58711	0.970945
1923	CHD3	1.301857	2.171205	0.029916	1.025997	1.651887
1935	RBM39	1.417844	2.606293	0.009153	1.090441	1.843549
1939	NCOA5	1.395293	2.381647	0.017235	1.060752	1.835342
1999	NCBP2	1.481274	3.434122	0.000594	1.183718	1.853627
2022	MRPL20	0.730221	-2.48776	0.012855	0.570003	0.935472
2036	POLR2B	1.504891	2.817627	0.004838	1.132481	1.999765
2068	PTPN12	1.447405	2.625851	0.008643	1.098307	1.907463

2131	BASP1	1.364409	2.497049	0.012523	1.069116	1.741263
2137	NPM3	0.63827	-3.56928	0.000358	0.498802	0.816734
2166	DHX15	1.36757	2.384652	0.017095	1.057332	1.768835
2174	CRKL	1.359401	2.19437	0.028209	1.033343	1.788343
2186	SYNCRIP	1.508537	2.952386	0.003153	1.148207	1.981946
2196	SSR1	1.390567	2.580091	0.009877	1.082471	1.786355
2208	NUDT21	1.296704	2.306607	0.021077	1.03982	1.617051
2215	ACIN1	1.317992	1.962542	0.049699	1.000363	1.736474
2233	PPP1R10	1.286656	2.051941	0.040175	1.011362	1.636887
2256	ADD1	1.37674	2.358855	0.018331	1.055554	1.795657
2267	RPL7L1	1.841653	4.133383	3.57E-05	1.378643	2.460163
2309	HNRNPH3	1.481381	2.520728	0.011711	1.091357	2.01079
2318	ZFR	1.527774	2.92783	0.003413	1.15039	2.028957
2323	SEC63	1.494289	3.486726	0.000489	1.192288	1.872786
2326	NOP58	1.445077	2.637807	0.008344	1.099227	1.899742
2397	YTHDF3	1.333811	2.353418	0.018602	1.049334	1.695411
2427	RTF1	1.337777	2.18205	0.029106	1.030062	1.737418
2515	UPF1	1.502373	2.763156	0.005725	1.125604	2.005257
2519	SDAD1	1.583856	3.619737	0.000295	1.234743	2.031678
2520	BCLAF1	1.531162	3.04794	0.002304	1.164244	2.013715

2527	RBM47	1.376448	2.525982	0.011538	1.07422	1.763708
2651	USP10	1.319309	2.280387	0.022585	1.039705	1.674107
2659	SSB	1.463513	2.643909	0.008195	1.103534	1.940917
2660	GOLGA4	1.401871	2.4779	0.013216	1.073162	1.831262
2665	PRRC2B	1.386297	2.320825	0.020296	1.0521	1.82665
2714	AFF4	1.435216	2.67752	0.007417	1.101673	1.869742
2725	CRNKL1	1.328303	2.40334	0.016246	1.053771	1.674357
2767	SAMD4B	1.435327	2.273096	0.02302	1.051044	1.960113
2785	TFB2M	1.336192	2.411768	0.015875	1.055795	1.691057
2847	CCAR1	1.531047	2.724377	0.006442	1.12695	2.080044
2848	NUFIP2	1.341685	2.363642	0.018096	1.05148	1.711987
2881	CAND1	1.263397	2.170013	0.030006	1.022889	1.560455
2977	CNOT1	1.283398	1.966154	0.049281	1.000786	1.645817
3022	PRPF40A	1.33539	2.254147	0.024187	1.038467	1.717211
3040	SNRPD3	1.412857	2.606907	0.009136	1.089555	1.832091
3056	ZRANB2	1.295586	2.053865	0.039989	1.01191	1.658787
3079	YTHDC1	1.328158	2.16998	0.030008	1.027847	1.716212
3108	THUMPD1	1.341957	2.144896	0.031961	1.025684	1.755754
3140	NXF1	1.322438	2.094977	0.036173	1.018174	1.717626

3181	PATL1	1.414658	2.282295	0.022472	1.050211	1.905575
3218	SLTM	1.299645	2.761608	0.005752	1.079049	1.565339
3226	PDS5A	1.43326	2.816213	0.004859	1.115654	1.841284
3248	CELF1	1.252115	2.033795	0.041972	1.008195	1.555047
3284	RARS2	1.336378	2.159387	0.03082	1.02714	1.738717
3392	DHX29	1.344723	2.185641	0.028842	1.031055	1.753815
3399	G3BP2	1.338081	2.291286	0.021947	1.043012	1.716624
3405	FXR1	1.321766	2.218185	0.026542	1.033008	1.69124
3422	PHF10	1.493577	2.89204	0.003827	1.138025	1.960214
3441	ANKRD17	1.300711	2.093059	0.036344	1.016859	1.6638
3465	UBFD1	1.328411	2.257858	0.023955	1.038179	1.69978
3470	PPID	1.423723	2.648055	0.008096	1.096143	1.849199
3487	GTPBP4	1.318833	2.194573	0.028194	1.030027	1.688617
3513	PRKAR2A	1.395068	2.335678	0.019508	1.055017	1.844725
3518	CDC5L	1.424802	2.661324	0.007783	1.097792	1.849222
3536	CSTF2T	1.319894	2.161766	0.030636	1.026248	1.697563
3614	CPSF6	1.258094	2.166261	0.030291	1.022106	1.548569
3639	ZNF638	1.382858	2.395348	0.016605	1.060689	1.802882
3641	MPHOSPH10	1.321416	2.072694	0.038201	1.015274	1.719872
3686	MRPL1	1.355057	2.183439	0.029004	1.031587	1.779956

3728	MRPL13	1.369359	2.550996	0.010742	1.075547	1.743434
3747	RPS15A	0.707862	-2.64242	0.008231	0.547837	0.914632
3757	RTCA	1.284482	1.980037	0.047699	1.002541	1.645712
3769	CEBPZ	1.423904	2.359582	0.018296	1.061679	1.909711
3771	ADD3	1.325295	2.229363	0.02579	1.034619	1.697636
3783	PURB	1.347881	2.647655	0.008105	1.080625	1.681233
3795	ELOF1	0.776944	-2.00873	0.044565	0.607352	0.993891
3805	SUGP2	1.418889	2.657419	0.007874	1.096175	1.836611
3813	ABCE1	1.328775	2.124192	0.033654	1.02222	1.727263
3820	PRPF4B	1.357275	2.44552	0.014464	1.06253	1.733783
3853	RBBP5	1.304412	2.035201	0.041831	1.009873	1.684857
3872	PPIL4	1.368027	2.273824	0.022977	1.044204	1.792273
3950	AFDN	1.53824	3.395001	0.000686	1.199647	1.972398
3978	EIF2AK2	1.382892	2.666581	0.007663	1.089701	1.754967
3992	NOL6	1.464674	2.633022	0.008463	1.102471	1.945876
4022	TFAM	1.289482	2.020593	0.043322	1.007658	1.650129
4023	NCBP1	1.428152	2.734068	0.006256	1.10617	1.843856
4055	RRP1B	1.285471	2.228082	0.025875	1.030681	1.603248
4190	PNO1	1.297996	2.069654	0.038485	1.013919	1.661665

4207	G3BP1	1.491192	2.834825	0.004585	1.131239	1.965679
4211	EIF4G3	1.509164	2.841279	0.004493	1.136164	2.004619
4298	SCAF11	1.361444	2.350926	0.018727	1.052651	1.760821
4364	ZC3H13	1.402013	2.442341	0.014592	1.069016	1.838736
4381	RBFOX2	1.288611	1.987588	0.046857	1.00353	1.654678
4411	FUBP3	1.586756	3.080598	0.002066	1.182878	2.128532
4526	SCAF8	1.597805	3.20687	0.001342	1.199872	2.127712
4534	HARS2	1.344281	2.252464	0.024293	1.039167	1.73898
4542	RSRC2	1.33122	2.235071	0.025413	1.035842	1.710828
4552	RPRD1B	1.286231	2.473959	0.013362	1.053689	1.570094
4559	LARP4	1.297394	2.219408	0.026459	1.030903	1.632774
4567	SRSF10	1.355317	2.200852	0.027746	1.033837	1.776762
4608	DROSHA	1.410964	2.761286	0.005757	1.105069	1.801534
4634	TBL2	1.276083	2.084556	0.03711	1.014678	1.604833
4703	NSRP1	1.41167	2.540902	0.011057	1.082017	1.841756
4707	EBP	0.779494	-1.96861	0.048998	0.608276	0.998907
4724	LARP7	1.392465	2.412389	0.015848	1.064059	1.822229
4770	SPAG9	1.340656	2.136631	0.032628	1.024536	1.754315
4818	NOP14	1.362367	2.475549	0.013303	1.066521	1.740278
4918	ZNF207	1.320152	2.167703	0.030181	1.026975	1.697024

4984	PPIG	1.501727	3.172012	0.001514	1.168091	1.930659
4995	RBBP6	1.493317	2.950814	0.003169	1.144138	1.949063
5025	ZC3H7A	1.428082	2.571188	0.010135	1.088399	1.873778
5047	RPF2	1.523767	2.997259	0.002724	1.156924	2.006931
5068	MACF1	1.398671	2.656872	0.007887	1.091998	1.79147
5089	LARP4B	1.39081	2.661222	0.007786	1.090819	1.773305
5094	PNISR	1.485857	2.888608	0.00387	1.135764	1.943864
5106	CSTF2	1.232458	2.051522	0.040216	1.009372	1.50485
5114	TSR1	1.388821	2.582066	0.009821	1.082351	1.78207
5136	SCAF4	1.53496	2.695426	0.00703	1.124029	2.096122
5167	HDAC2	1.452327	2.973581	0.002943	1.135647	1.857313
5189	MARK2	1.426198	2.747386	0.006007	1.107106	1.837259
5204	FNDC3B	1.332262	2.128967	0.033257	1.023034	1.734957
5207	RDX	1.420974	2.452616	0.014182	1.073124	1.88158
5218	ZNF106	1.40776	2.590524	0.009583	1.086809	1.823491
5274	DIDO1	1.271877	2.216856	0.026633	1.028261	1.57321
5284	SECISBP2L	1.51883	3.196226	0.001392	1.175454	1.962515
5302	ESF1	1.421004	2.647909	0.008099	1.095583	1.843085
5393	AIMP1	1.401106	2.723221	0.006465	1.099139	1.786032

5437	KDM5A	1.32334	2.212733	0.026916	1.032521	1.696071
5460	DCAF13	1.320697	2.514133	0.011933	1.063231	1.640509
5700	BRIX1	1.355765	2.356975	0.018425	1.052605	1.74624
5704	ZCCHC7	1.300708	2.006491	0.044804	1.006115	1.681557
5729	DNAJC21	1.391453	2.669954	0.007586	1.09182	1.773316
5742	ROCK2	1.308442	2.185914	0.028822	1.028178	1.6651
5789	MRPS9	1.429909	2.587901	0.009656	1.090648	1.874701
5829	TRUB2	1.41912	2.62047	0.008781	1.092238	1.84383
5949	NAA15	1.333831	2.324386	0.020105	1.046197	1.700544
5965	FMR1	1.468391	3.22452	0.001262	1.162599	1.854612
6024	GPATCH8	1.490977	2.653943	0.007956	1.110097	2.00254
6091	STAU2	1.368873	2.783416	0.005379	1.097342	1.707593
6093	KMT2C	1.301211	1.986269	0.047003	1.003493	1.687256
6196	MDC1	1.269751	1.979986	0.047705	1.002418	1.60838
6215	TCERG1	1.377833	2.340061	0.019281	1.05344	1.802119
6223	SMNDC1	1.337626	2.21396	0.026832	1.033936	1.730516
6236	HLTF	1.342487	2.202262	0.027647	1.032935	1.744806
6290	SMG1	1.464051	2.88655	0.003895	1.13017	1.89657
6293	FAM98B	1.447569	2.686374	0.007223	1.105192	1.896011
6317	UTP23	1.491302	3.325702	0.000882	1.178357	1.887359

6335	SECISBP2	1.644128	3.508455	0.000451	1.24539	2.17053
6346	MSI2	1.320518	2.252767	0.024274	1.036797	1.68188
6368	IREB2	1.291728	2.009427	0.044492	1.006321	1.658082
6404	R3HDM2	1.438409	2.752939	0.005906	1.110395	1.86332
6507	EDC4	1.28652	1.983541	0.047307	1.002999	1.650185
6530	GIGYF2	1.353115	2.302339	0.021316	1.045997	1.750407
6565	GNE	1.36179	2.455462	0.01407	1.064297	1.742439
6612	RC3H2	1.399913	2.880458	0.003971	1.113496	1.760003
6639	PTCD3	1.371395	2.401219	0.016341	1.059755	1.774678
6661	NOL8	1.354084	2.458714	0.013944	1.063419	1.724198
6674	SRPK1	1.27098	2.10798	0.035033	1.01698	1.588419
6709	SPATS2	1.268907	1.98198	0.047481	1.002649	1.605872
6723	EXOSC2	1.388982	2.576552	0.009979	1.081803	1.783383
6826	RBM23	1.266736	2.013095	0.044105	1.00626	1.594638
6909	PHF3	1.402542	2.522608	0.011649	1.078371	1.824163
6932	ASCC3	1.351661	2.28891	0.022085	1.044257	1.749558
6988	TNRC6A	1.424314	2.532588	0.011322	1.083255	1.872755
7007	CHERP	1.383884	2.143804	0.032049	1.028253	1.862515
7036	UBAP2	1.384467	2.416117	0.015687	1.063344	1.802569

7126	RWDD4	1.275212	1.966423	0.04925	1.000799	1.624868
7150	PHF6	1.467101	2.96402	0.003036	1.138644	1.890306
7152	UPF3B	1.354579	2.456715	0.014021	1.063288	1.725669
7411	DHX33	1.486231	3.033759	0.002415	1.150561	1.91983
7424	ZFC3H1	1.534695	3.468048	0.000524	1.204736	1.955024
7425	HELZ	1.381214	2.352875	0.018629	1.055413	1.807588
7577	METTL16	1.543507	3.373677	0.000742	1.19948	1.986204
7617	SRFBP1	1.304331	2.013231	0.04409	1.007055	1.689362
7685	AGO2	1.478738	3.013546	0.002582	1.146559	1.907154
7720	CDC40	1.593895	3.36786	0.000758	1.215167	2.09066
7724	RIF1	1.380554	2.547851	0.010839	1.077248	1.769258
7760	UTP20	1.293726	2.168863	0.030093	1.025114	1.632722
7771	YTHDC2	1.377537	2.342348	0.019163	1.053679	1.800936
7806	SREK1	1.35516	2.251561	0.02435	1.040145	1.765579
7861	URB2	1.286111	1.962359	0.049721	1.000307	1.653573
7869	RRP15	1.311506	2.185417	0.028858	1.02837	1.672595
7940	CNOT4	1.331899	2.03146	0.042208	1.010138	1.756151
8004	URB1	1.367995	2.452148	0.014201	1.064913	1.757335
8056	HMGN5	1.515417	3.36831	0.000756	1.189826	1.930105
8118	GNL3L	1.49706	3.301176	0.000963	1.17814	1.902312

8172	ROR2	1.54942	2.871997	0.004079	1.149186	2.089047
8255	RBM27	1.34041	2.273611	0.022989	1.041244	1.725531
8271	RC3H1	1.358501	2.235088	0.025412	1.038434	1.777219
8294	MECP2	1.610855	4.379698	1.19E-05	1.301357	1.99396
8320	CLASP2	1.300483	1.986462	0.046982	1.003511	1.685338
8322	SNTB2	1.321125	2.240573	0.025054	1.035493	1.685546
8359	STX7	1.510039	3.028414	0.002458	1.156508	1.97164
8471	TRNAU1AP	0.756312	-2.13186	0.033018	0.585036	0.977731
8493	FASTKD3	1.547267	3.284416	0.001022	1.192457	2.007649
8562	DDX55	1.311867	2.045889	0.040767	1.011466	1.701485
8596	PURA	1.480265	3.006568	0.002642	1.146294	1.911538
8673	DDX52	1.540163	3.186787	0.001439	1.180886	2.008747
8779	ZMAT3	1.350946	2.42913	0.015135	1.059819	1.722045
8972	NCBP3	1.40091	2.493246	0.012658	1.074771	1.826017
9005	ZCCHC8	1.317584	1.975474	0.048214	1.002168	1.732273
9020	UTP15	1.318954	2.159426	0.030817	1.025901	1.69572
9100	USP42	1.357243	2.579344	0.009899	1.076106	1.711827
9107	STXBP1	1.444696	2.786634	0.005326	1.115318	1.871348
9118	NAF1	1.289297	2.316338	0.02054	1.039868	1.598556

9133	MTPAP	1.337893	2.237254	0.02527	1.036738	1.726529
9136	NOM1	1.312371	2.009203	0.044516	1.006684	1.710883
9151	PMS1	1.404467	2.530388	0.011394	1.079577	1.827132
9163	ZCCHC9	1.348746	2.156306	0.03106	1.027616	1.770228
9276	FASTKD1	1.444729	2.936129	0.003323	1.130118	1.846925
9437	ADARB1	1.340955	2.205116	0.027446	1.033154	1.740457
9503	CDK13	1.344224	2.260618	0.023783	1.040127	1.737229
9550	ATXN2	1.459741	2.820443	0.004796	1.122324	1.898599
9585	RBM12B	1.614175	3.317931	0.000907	1.216495	2.141859
9672	NKRF	1.431738	2.862913	0.004198	1.119847	1.830494
9713	POP1	1.442137	3.121225	0.001801	1.145932	1.814905
9801	EIF4E	1.386776	2.678946	0.007385	1.091722	1.761574
9872	NKAP	1.465842	3.54014	0.0004	1.186136	1.811506
9986	RBM45	1.422441	2.636018	0.008389	1.094582	1.848502
9990	ZNF346	1.373395	2.760056	0.005779	1.096338	1.720466
10007	FAM120C	1.24482	1.996796	0.045847	1.004048	1.543329
10180	PRKRA	1.333651	1.998949	0.045614	1.005631	1.768665
10292	GTPBP10	1.315127	2.140899	0.032282	1.023421	1.689978
10313	TNRC6B	1.355535	2.231979	0.025616	1.037769	1.770603
10418	FAM133B	1.508838	3.016114	0.00256	1.154928	1.971197

10458	METTL8	1.317579	2.11932	0.034063	1.020954	1.700385
10554	LLPH	1.237418	2.088187	0.036781	1.013167	1.511305
10569	TRMT10A	1.336478	2.281547	0.022516	1.041728	1.714625
10611	TFB1M	1.325655	2.160122	0.030763	1.026466	1.712049
10702	ZC3H8	1.36114	2.366043	0.017979	1.054342	1.757212
10823	MSI1	1.297815	2.0962	0.036064	1.017087	1.656027
10901	TEFM	1.396267	2.610414	0.009043	1.086732	1.793968
11070	MYEF2	1.316869	2.116823	0.034275	1.020606	1.699132
11251	MTO1	1.575073	3.423391	0.000618	1.214344	2.042958
11314	KHDRBS3	1.291214	2.081486	0.037389	1.015033	1.64254
11632	AGO3	1.348359	2.272192	0.023075	1.041926	1.744914
11941	C4BPA	0.613935	-2.59987	0.009326	0.425005	0.886851
11949	SRP19	1.4647	3.26552	0.001093	1.16484	1.841752
12084	PTCD2	1.489077	3.191785	0.001414	1.166097	1.901513
12458	ZC3H10	1.448192	2.837074	0.004553	1.121298	1.870386
14197	DMGDH	1.329242	2.685206	0.007249	1.079901	1.636155
14559	RIMS1	1.304215	2.408479	0.016019	1.050705	1.618892
14661	PCSK9	0.669292	-2.40567	0.016143	0.482547	0.928306
15301	PLA2G1B	0.706828	-2.40887	0.016002	0.532978	0.937387

15884	ADARB2	1.173437	2.040859	0.041265	1.00636	1.368253
17642	PTRH1	1.238434	2.295963	0.021678	1.03179	1.486464
